# Effects and safety of acupuncture on inflammatory biomarkers in patients with coronary heart disease: a systematic review and meta-analysis

**DOI:** 10.3389/fcvm.2026.1891405

**Published:** 2026-10-09

**Authors:** Linghao Meng, Di Sun, Houfang Ma, Shuhan Zhao, Songyan Bai, Wenzeng Zhu, Limei Zhang, Shuai Shi

**Affiliations:** 1 Guang’anmen Hospital, China Academy of Chinese Medical Sciences, Beijing, China; 2 China Center for Evidence-Based Traditional Chinese Medicine, China Academy of Chinese Medical Sciences, Beijing, China

**Keywords:** acupuncture, coronary heart disease, inflammatory biomarkers, lipid metabolism, meta-analysis, systematic review

## Abstract

**Background:**

Inflammation contributes to the development and progression of coronary heart disease (CHD), and residual inflammatory risk remains an important clinical concern despite advances in standard cardiovascular therapy. Acupuncture interventions have been increasingly used as adjunctive therapies for CHD, but their effects on inflammatory biomarkers have not been systematically evaluated. This systematic review and meta-analysis aimed to assess the effects of acupuncture interventions on inflammatory biomarkers and to evaluate their safety in patients with CHD.

**Methods:**

PubMed, Embase, the Cochrane Library, Web of Science, CNKI, Wanfang, VIP, and SinoMed were searched from inception to 12 March 2026. Randomized controlled trials evaluating acupuncture interventions in patients with CHD were included. The co-primary outcomes were high-sensitivity C-reactive protein (hs-CRP) and C-reactive protein (CRP). Secondary inflammatory outcomes included interleukin-6 (IL-6) and tumor necrosis factor-alpha (TNF-α). Meta-analyses were performed using Review Manager 5.4 and Stata 18.0. Random-effects 95% prediction intervals were calculated for heterogeneous inflammatory outcomes with at least three studies. Subgroup analyses were conducted by clinical setting, and a *post hoc* exploratory analysis by comparator type was undertaken for CRP, with a formal test for interaction. The certainty of evidence was assessed using GRADE.

**Results:**

Thirteen randomized controlled trials involving 927 patients were included. The pooled estimates were lower for CRP standardized mean difference (SMD) = −0.87, 95% confidence interval (CI) −1.43 to −0.30; 95% prediction interval (PI) −2.15 to 0.42; low-certainty evidence] and IL-6 (SMD = −0.74, 95% CI −1.31 to −0.17; 95% PI −1.93 to 0.45; low-certainty evidence), but heterogeneity was substantial and both prediction intervals included no effect. For hs-CRP, the pooled estimate was not statistically significant (SMD = −0.79, 95% CI −1.65 to 0.07; 95% PI −2.99 to 1.42), and the evidence remained inconclusive. TNF-α also favored acupuncture, but heterogeneity and imprecision were substantial. Total cholesterol (TC) and triglycerides (TG) decreased, whereas low-density lipoprotein cholesterol (LDL-C) and high-density lipoprotein cholesterol (HDL-C) did not differ significantly. Reported adverse events did not differ significantly between groups, but safety reporting was sparse.

**Conclusion:**

The pooled data yielded lower average CRP and IL-6 estimates, but low-certainty evidence, substantial heterogeneity, and prediction intervals including no effect preclude inference of a consistent anti-inflammatory benefit. Findings for hs-CRP and TNF-α were inconclusive. Current studies cannot distinguish acupuncture-specific effects from non-specific or contextual effects, and evidence on cardiovascular events and safety remains limited. Further well-designed trials are needed.

**Systematic review registration:**

https://www.crd.york.ac.uk/PROSPERO/view/CRD420261279145.

## 1 Introduction

Coronary heart disease (CHD) remains one of the leading contributors to cardiovascular morbidity and mortality worldwide. According to the 2025 Heart Disease and Stroke Statistics update, an estimated 8.99 million deaths globally were attributable to ischemic heart disease in 2021 (1). In China, the burden of CHD is also substantial; the 2024 Annual Report on Cardiovascular Health and Diseases in China reported that the prevalence of adult CHD was 758 per 100,000 population (2). Clinically, CHD encompasses a broad spectrum of conditions, including acute coronary syndrome (ACS), chronic coronary syndrome (CCS), and post-percutaneous coronary intervention (PCI) status. Although antithrombotic therapy, lipid-lowering treatment, anti-ischemic medication, and revascularization strategies have improved substantially, many patients continue to experience recurrent ischemic symptoms, residual cardiovascular risk, and impaired quality of life (3–5).

Inflammation runs through the entire development and progression of CHD and can be understood from two complementary perspectives: chronic low-grade inflammation and acute inflammatory activation. Chronic inflammation contributes to the initiation and progression of atherosclerosis and plaque instability, involving endothelial dysfunction, leukocyte recruitment, foam-cell formation, and thinning of the fibrous cap (6, 7). Acute inflammatory activation is more prominent in plaque rupture, ACS, PCI-related vascular injury, and perioperative stress, and may further amplify thrombosis, microvascular dysfunction, and myocardial injury. Randomized controlled trials targeting inflammatory pathways, such as CANTOS, COLCOT, and LoDoCo2, further demonstrated that anti-inflammatory strategies can reduce cardiovascular events in selected patients with established atherosclerotic cardiovascular disease (8–10). These findings suggest that inflammation is not only a marker of cardiovascular risk but also a potential therapeutic target.

Lipid metabolism and inflammation are closely interconnected in the development and progression of CHD. Low-density lipoprotein cholesterol (LDL-C) has a causal role in atherosclerotic cardiovascular disease, and lipid-lowering therapy remains a cornerstone of secondary prevention (11, 12). However, in real-world practice, optimal long-term risk-factor control may be limited by age, comorbidities, poor adherence, and drug intolerance (13). Therefore, safe and accessible non-pharmacological adjunctive interventions may be clinically meaningful, particularly in patients with ACS, post-PCI status, or perioperative stress, in whom inflammatory activation and metabolic disturbance often coexist.

Acupuncture has been investigated as an adjunctive non-pharmacological treatment for CHD. Previous clinical studies and evidence syntheses have mainly focused on angina symptoms, electrocardiographic response, or glycolipid metabolism (14–18), while experimental research suggests that acupuncture may influence inflammatory signaling through neuroimmune pathways (19, 20). Accessible adjunctive approaches may also be relevant when long-term risk-factor control is complicated by comorbidity, adherence, or treatment intolerance (13). However, the effects of acupuncture interventions on inflammatory biomarkers in patients with CHD have not been systematically synthesized.

Therefore, this systematic review and meta-analysis aimed to evaluate the effects and safety of acupuncture interventions on inflammatory biomarker levels in patients with CHD.

## 2 Methods

### 2.1 Study registration and reporting

This systematic review and meta-analysis was registered in PROSPERO (CRD420261279145). The eligibility criteria, outcome hierarchy, and principal analytical methods were specified before quantitative synthesis. Additional analyses undertaken to explore heterogeneity are described as *post hoc* exploratory analyses. The review was conducted and reported in accordance with the Preferred Reporting Items for Systematic Reviews and Meta-Analyses (PRISMA) 2020 statement (21), and the completed checklist is provided in Supplementary Table 1.

### 2.2 Search strategy

A systematic literature search was conducted in PubMed, Embase, the Cochrane Library, Web of Science, CNKI, Wanfang, VIP, and SinoMed from database inception to 12 March 2026. The language was restricted to Chinese and English. The search terms drew on previous evidence syntheses of acupuncture-related interventions for CHD (15–18) and were adapted to the indexing and syntax of each database. Controlled vocabulary and free-text terms were combined for CHD, acupuncture interventions, and randomized trials. Inflammation-related terms were not required in the core strategy because individual biomarkers were inconsistently indexed and reported. The complete database-specific strategies are provided in the Supplementary Material. Reference lists of the included studies and relevant reviews were also screened.

### 2.3 Eligibility criteria

Studies were included if they met the following criteria:

(1) participants diagnosed with CHD, including ACS, CCS, post-PCI status, or CHD undergoing non-cardiac surgery;
(2) the experimental group received acupuncture interventions, including manual acupuncture, electroacupuncture, transcutaneous electrical acupoint stimulation, or true acupuncture, either alone or in combination with conventional treatment;
(3) the control group received conventional treatment, blank control, sham acupuncture, or sham electroacupuncture;
(4) at least one inflammation-related outcome was reported;
(5) randomized controlled trials.

Studies were excluded if they were non-randomized studies, animal experiments, case reports, reviews, study protocols, duplicate publications, or studies with incomplete or unavailable outcome data.

### 2.4 Outcomes

The co-primary outcomes were hs-CRP and CRP, which were analyzed separately because the primary studies reported them as distinct measures. Secondary inflammatory outcomes included IL-6 and TNF-α. Other inflammation-related biomarkers, including neutrophil count, neutrophil-to-lymphocyte ratio (NLR), IL-10, IL-1β, and intercellular adhesion molecule-1 (ICAM-1), were summarized narratively when reported by only one study. Secondary non-inflammatory outcomes included lipid metabolism indicators (LDL-C, HDL-C, TG, and TC), electrocardiographic response, and adverse events.

### 2.5 Literature management and data extraction

Zotero 8.0.5 was used for literature management. Two reviewers independently screened titles, abstracts, and full texts and extracted data using a predefined form. Extracted information included first author, publication year, country, disease type, sample size, sex, age, intervention and control measures, acupoint selection, treatment frequency, treatment duration, stimulation or needle-retention time, and reported outcomes. Where reported, details of background treatment, sham-control procedures, and clinical endpoints were also extracted. Disagreements were resolved through discussion or consultation with a third reviewer.

### 2.6 Risk of bias assessment

The revised Cochrane Risk of Bias tool for randomized trials (RoB 2) was used to assess methodological quality (22). The following domains were evaluated: bias arising from the randomization process, bias due to deviations from intended interventions, bias due to missing outcome data, bias in measurement of the outcome, and bias in selection of the reported result. The overall risk of bias for each study was judged as low risk, some concerns, or high risk.

### 2.7 Data synthesis and statistical analysis

Meta-analyses were performed using Review Manager 5.4 and Stata 18.0. Trials were quantitatively synthesized when they enrolled patients with CHD, evaluated eligible acupuncture interventions and control conditions, and reported the same outcome with extractable data. For continuous outcomes, the mean difference (MD) was used when the same scale and unit were reported; the standardized mean difference (SMD) was used when inflammatory biomarkers were reported using different units, assay methods, or data scales that could not be reliably harmonized. Risk ratios (RRs) were calculated for dichotomous outcomes. All estimates were reported with 95% confidence intervals (CIs). Statistical heterogeneity was evaluated using the *χ*^2^ test, I^2^, and *τ*^2^. Because clinical setting, intervention modality, comparator design, and treatment regimen varied, a random-effects model was used when substantial heterogeneity was present; otherwise, a fixed-effect model was applied. For inflammatory outcomes with at least three studies and I^2^ ≥ 50%, 95% prediction intervals (PIs) were calculated under the random-effects model. Subgroup analyses by clinical setting were planned before quantitative synthesis. A *post hoc* analysis by comparator type was undertaken when at least two studies were available in each subgroup, with a formal test for interaction. Potential analyses by intervention modality, treatment duration, and baseline inflammatory status were assessed for feasibility but were not undertaken when the available studies could not form comparable subgroups. Meta-regression was considered when at least 10 studies were available for a potential effect modifier and the relevant study-level covariates were sufficiently comparable. Leave-one-out sensitivity analyses were conducted for outcomes with I^2^ ≥ 50%. Outcomes reported by fewer than two studies were summarized narratively.

### 2.8 Certainty of evidence

The certainty of evidence for each outcome was assessed using the Grading of Recommendations Assessment, Development and Evaluation (GRADE) approach (23) and rated as high, moderate, low, or very low. Risk of bias was judged from the RoB 2 assessments. Inconsistency was assessed by considering I^2^, *τ*^2^, overlap of study CIs, variation in effect magnitude or direction, clinical heterogeneity, and prediction intervals where available. Indirectness considered differences in populations, interventions, comparators, and the relationship between biomarker outcomes and clinical cardiovascular outcomes. Imprecision was assessed from the total information size, the width of the pooled CI, and whether the CI crossed the null or encompassed materially different effects. No validated minimally important difference was available for the standardized biomarker outcomes; therefore, no formal optimal information size threshold based on an assumed minimally important difference was applied. The available information size and the pooled CI were considered together. Prediction intervals were considered principally in the assessment of inconsistency and were not used alone to downgrade imprecision. Potential publication bias was assessed qualitatively for each outcome by considering the number and size of studies, publication type, study setting, and the possibility of selective outcome reporting. Funnel plots and regression-based tests were planned only for outcomes with at least 10 studies; when this threshold was not met, the absence of a formal test was not interpreted as evidence that publication bias was absent. The reasons for each domain judgment are provided in the Supplementary Material.

## 3 Results

### 3.1 Study selection

A total of 1,046 records were identified through database searching. After removing 280 duplicates, 766 records remained for title and abstract screening, of which 598 were excluded. Full texts were sought for 168 reports, and 34 reports could not be retrieved. A total of 134 full-text reports were assessed for eligibility. Of these, 121 were excluded because of inconsistent interventions, inconsistent outcomes, non-randomized design, or unavailable extractable data. Finally, 13 randomized controlled trials were included in the systematic review and meta-analysis. The flow chart of study screening is presented in Figure 1.

**Figure 1:**
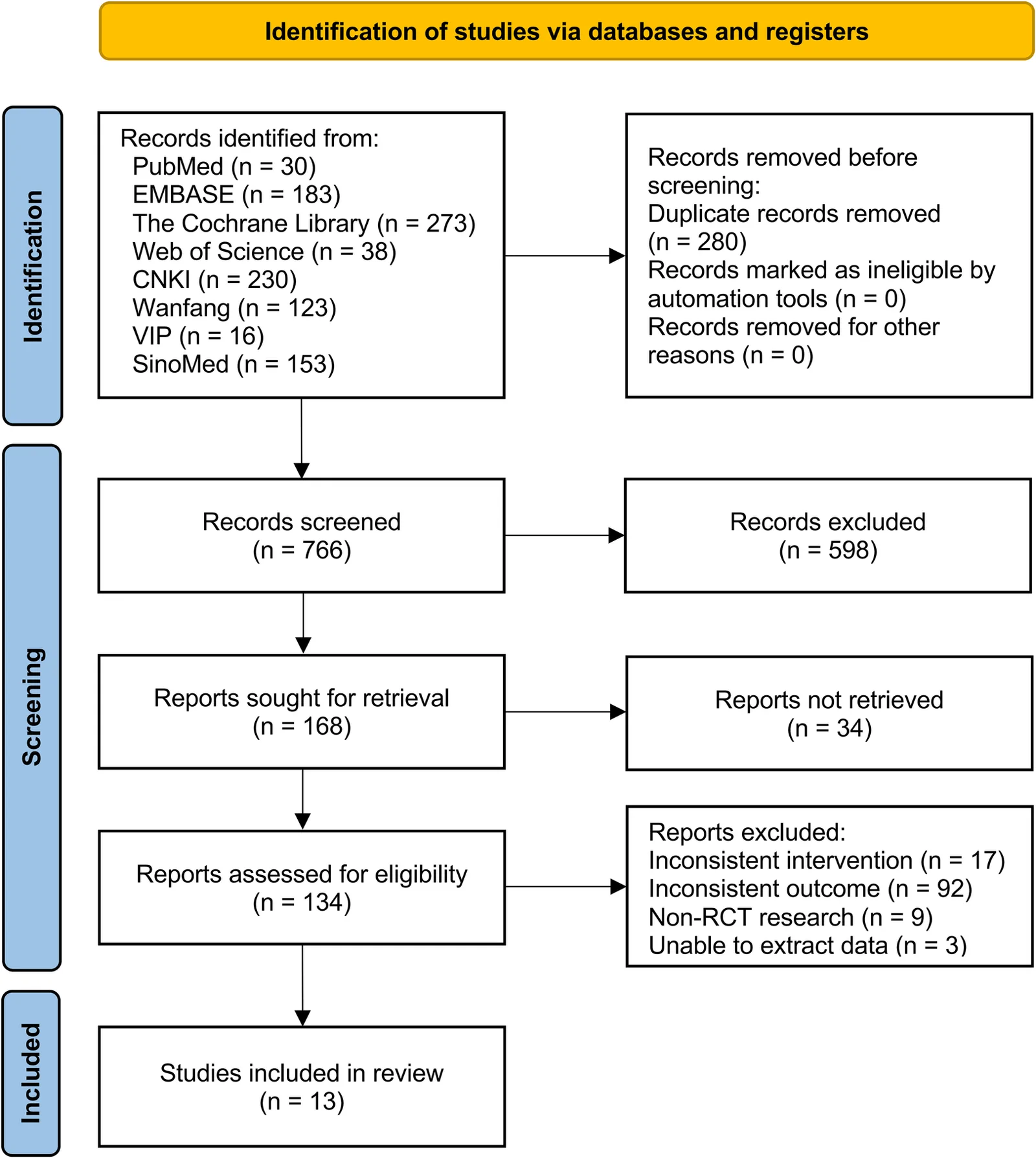
PRISMA flow diagram.

### 3.2 Characteristics of included studies

All 13 included studies were conducted in China, enrolling a total of 927 patients with CHD, including 469 participants in the experimental groups and 458 in the control groups (24–36). The trials involved patients with CHD in different clinical settings, including ACS, CCS, post-PCI status, and CHD undergoing non-cardiac surgery. The sample size of individual studies ranged from 30 to 122 participants. The acupuncture interventions included manual acupuncture, electroacupuncture, and transcutaneous electrical acupoint stimulation, either alone or combined with conventional treatment. Control interventions included conventional treatment, blank control, non-penetrating sham acupuncture, inactive-electrode control, and zero-current sham TEAS. Treatment duration ranged from a single perioperative session to 12 weeks. Reported outcomes included inflammatory biomarkers, lipid metabolism indicators, electrocardiographic response, and adverse events; two studies reported major adverse cardiovascular events (MACE). The details provided for background therapy and biomarker assessment varied across reports. Study characteristics are shown in Table 1, and additional extracted information is provided in Supplementary Table 7.

**Table 1:** Characteristics of included studies.

Study	Country	Patient Disease Type	Sample (EG/CG)	Intervention		Sex (male/female)	Mean age (EG/CG, years)	Acupoints	Treatment frequency	Treatment duration	Stimulation/needle-retention time	Reported outcomes
				EG	CG							
Xu (24)	China	CHD, ACS	30/20	EA + CT	CT	EG: 18/12 CG: 11/9	EG: 61.83 ± 8.38 CG: 62.13 ± 9.15	ST40	Once daily	30 days	30 min	hs-CRP, LDL-C, HDL-C, TG, TC, AEs
He (2013	China	CHD, CCS	40/40	A	BC	EG: 20/20 CG: 22/18	EG: 65.2 ± 12.3 CG: 65.6 ± 11.3	SP4, PC6, GB41, TE5, LU7, KI6, SI3, BL62	Once daily	2 weeks	30 min	hs-CRP, ECG response, AEs
Lyu (26)	China	CHD, ACS	54/54	A + CT	CT	EG: 44/10 CG: 45/9	EG: 61.89 ± 6.44 CG: 60.04 ± 5.99	PC6, HT7, HT6, Chest pain point	Once daily	1 week	30 min	hs-CRP
Wang et al. (27)	China	CHD, CCS	15/15	EA + CT	CT	EG: 7/8 CG: 6/9	EG: 59.8 ± 3.2 CG: 58.8 ± 4.3	PC6	Once every other day	4 weeks	30 min	NEUT, NLR
Li et al. (28)	China	CHD + NCS	61/61	TEAS	Sham TEAS	EG: 31/30 CG: 33/28	EG: 68.5 ± 6.3 CG: 67.9 ± 5.8	PC6, PC4	One session	Perioperative period	Not applicable; stimulation from 30 min before induction to the end of surgery	CRP
Zhang (29)	China	CHD, post-PCI	30/30	A + CT	CT	EG: 17/13 CG: 16/14	EG: 63.27 ± 8.79 CG: 61.03 ± 8.10	LU7, LI4, HT7, PC6, LI11, LU5	Five times per week	4 weeks	45 min	hs-CRP, IL-6, LDL-C, HDL-C, TG, TC, ECG response, MACE (3-month follow-up)
Zhou et al. (30)	China	CHD + NCS	40/40	EA + CT	CT	EG: 26/14 CG: 23/17	EG: 67 ± 8 CG: 69 ± 8	Neima point, PC6	Two sessions	Perioperative period	30 min	IL-6, TNF-α
Deng et al. (31)	China	CHD, ACS	34/34	A + CT	CT	EG: 20/14 CG: 18/16	EG: 50.87 ± 10.23 CG: 52.94 ± 11.52	CV17, BL15, PC6, ST36, SP6	Once daily	4 weeks	30 min	hs-CRP, LDL-C, HDL-C, TG, TC
Zhang et al. (32)	China	CHD, post-PCI	16/16	A + CT	CT	EG: 6/10 CG: 8/8	EG: 69.50 ± 11.6 CG: 67.1 ± 14.7	ACU1, ACU2	Once daily	2 weeks	During 60-min exercise; removed after 5-min observation	CRP, AEs, MACE (2-, 4-, and 8-week follow-up)
Zhan et al. (33)	China	CHD + NCS	52/52	EA + CT	CT	EG: 30/22 CG: 28/24	EG: 60.94 ± 5.61 CG: 62.11 ± 5.32	ST36, PC6, GB34, GB24, ST30	One session	Perioperative period	30 min	CRP, IL-6, TNF-α, ICAM-1
Ling (34)	China	CHD, post-PCI	42/42	EA + CT	Sham EA + CT	EG: 36/6 CG: 32/10	EG: 60.17 ± 13.18 CG: 60.79 ± 13.79	PC6, HT6, ST36	Once daily	5 days	30 min	CRP, IL-6, TNF-α, IL-10, IL-1β, AEs
Li (35)	China	CHD, CCS	25/24	TEAS + CT	CT	EG: 9/16 CG: 9/15	EG: 62.84 ± 9.74 CG: 59.13 ± 11.32	PC6, ST36	Three times per week	12 weeks	20 min	hs-CRP, AEs
Zeng (36)	China	CHD, ACS	30/30	EA + CT	CT	EG: 18/12 CG: 13/17	EG: 62.7 ± 8.84 CG: 64.9 ± 8.84	PC6, HT6, SP10, LR3, SP6, ST36	Once daily	2 weeks	20 min	CRP, LDL-C, HDL-C, TG, TC, ECG response, AEs

EG, experimental group; CG, control group; CHD, coronary heart disease; ACS, acute coronary syndrome; CCS, chronic coronary syndrome; NCS, non-cardiac surgery; PCI, percutaneous coronary intervention; post-PCI, after percutaneous coronary intervention; A, acupuncture; EA, electroacupuncture; TEAS, transcutaneous electrical acupoint stimulation; CT, conventional treatment; BC, blank control; SA, sham acupuncture; SEA, sham electroacupuncture; hs-CRP, high-sensitivity C-reactive protein; CRP, C-reactive protein; IL-6, interleukin-6; IL-10, interleukin-10; IL-1β, interleukin-1 beta; TNF-α, tumor necrosis factor-alpha; ICAM-1, intercellular adhesion molecule-1; LDL-C, low-density lipoprotein cholesterol; HDL-C, high-density lipoprotein cholesterol; TG, triglycerides; TC, total cholesterol; ECG, electrocardiogram; AEs, adverse events; NEUT, neutrophils; NLR, neutrophil-to-lymphocyte ratio; ACU1, upper-jiao area; ACU2, heart area; MACE, major adverse cardiovascular events; NR, not reported. Additional available information on background treatment, sham controls, and cardiovascular endpoints is provided in Supplementary Table 7.

### 3.3 Risk of bias

The results of the risk-of-bias assessment are shown in Figures 2 and 3. Overall, most included studies were judged as having some concerns. The main methodological concerns were insufficient reporting of allocation concealment, lack of blinding, and absence of prespecified analysis plans. The risk of bias in outcome measurement was generally low because most outcomes were objective laboratory indicators. Two studies were judged as having high overall risk of bias (28, 30).

**Figure 2:**
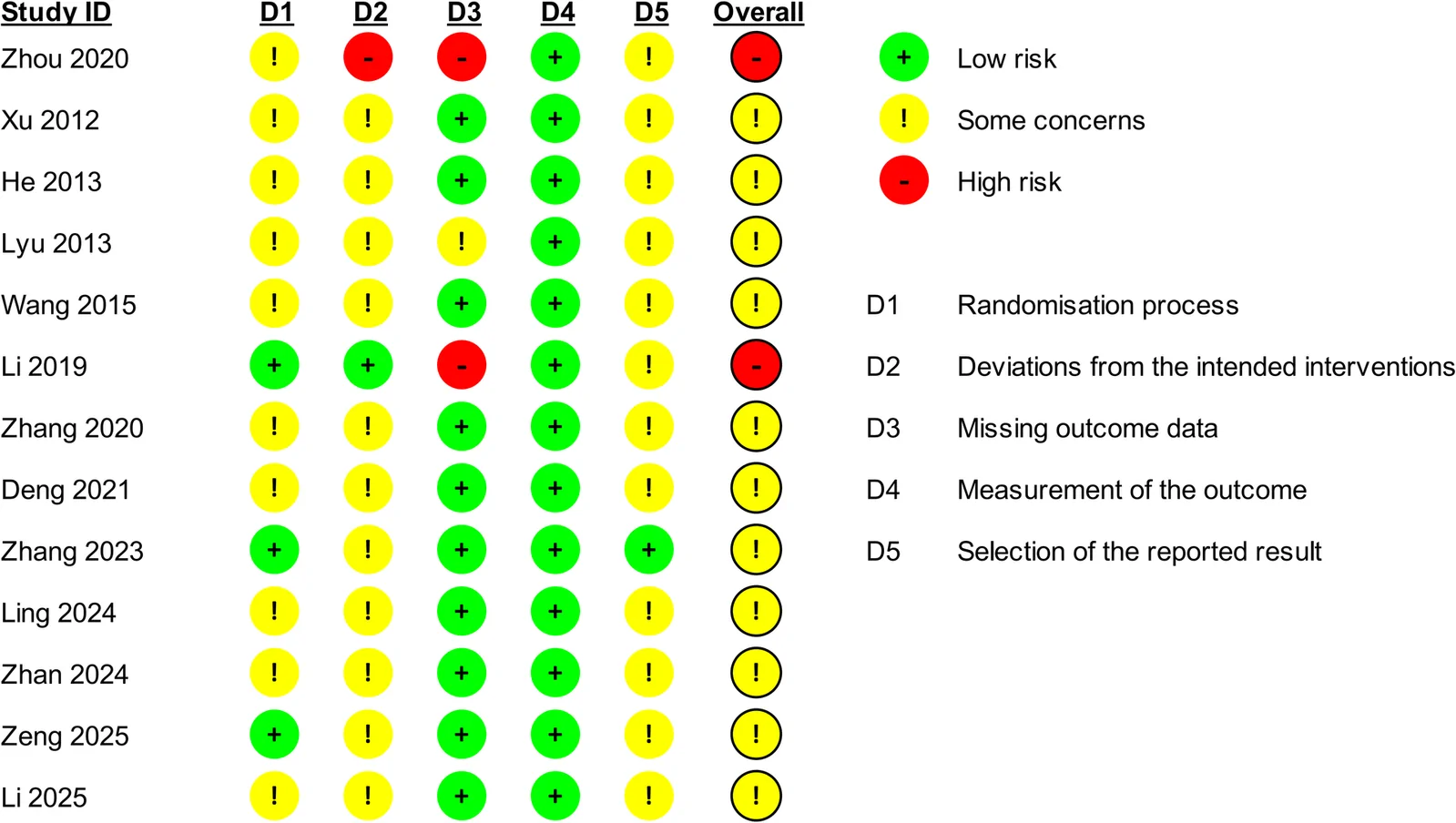
Risk-of-bias assessment of the included studies.

**Figure 3:**
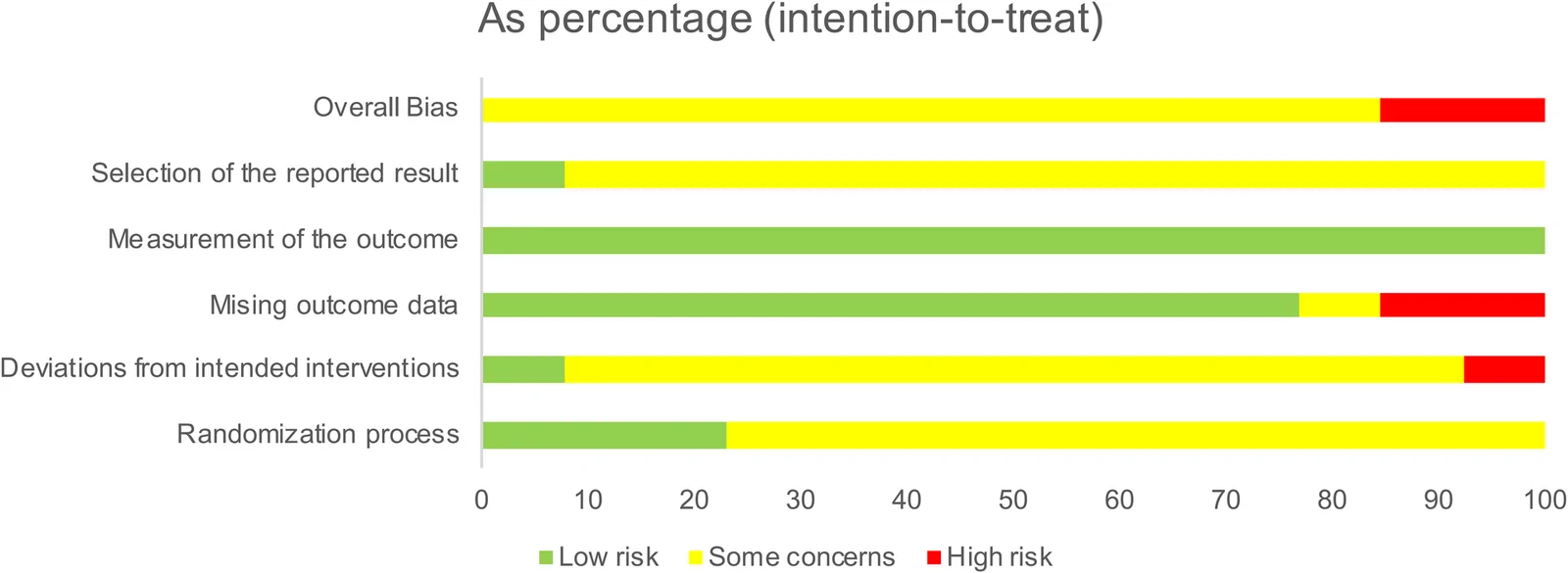
Summary of the RoB 2 assessment.

### 3.4 Inflammatory biomarkers

#### 3.4.1 hs-CRP

Six studies reported hs-CRP (24–26, 29, 31, 35). The pooled estimate favored acupuncture interventions but did not reach statistical significance (SMD = −0.79, 95% CI −1.65 to 0.07, *P* = 0.07; I^2^ = 94%; *τ*^2^ = 1.08). The 95% PI ranged from −2.99 to 1.42, crossing the null and including effects in both directions. Therefore, the evidence for hs-CRP is inconclusive rather than confirmatory. No significant interaction was observed by clinical setting (P for interaction = 0.39; Figure 4). The certainty of evidence was very low.

**Figure 4:**
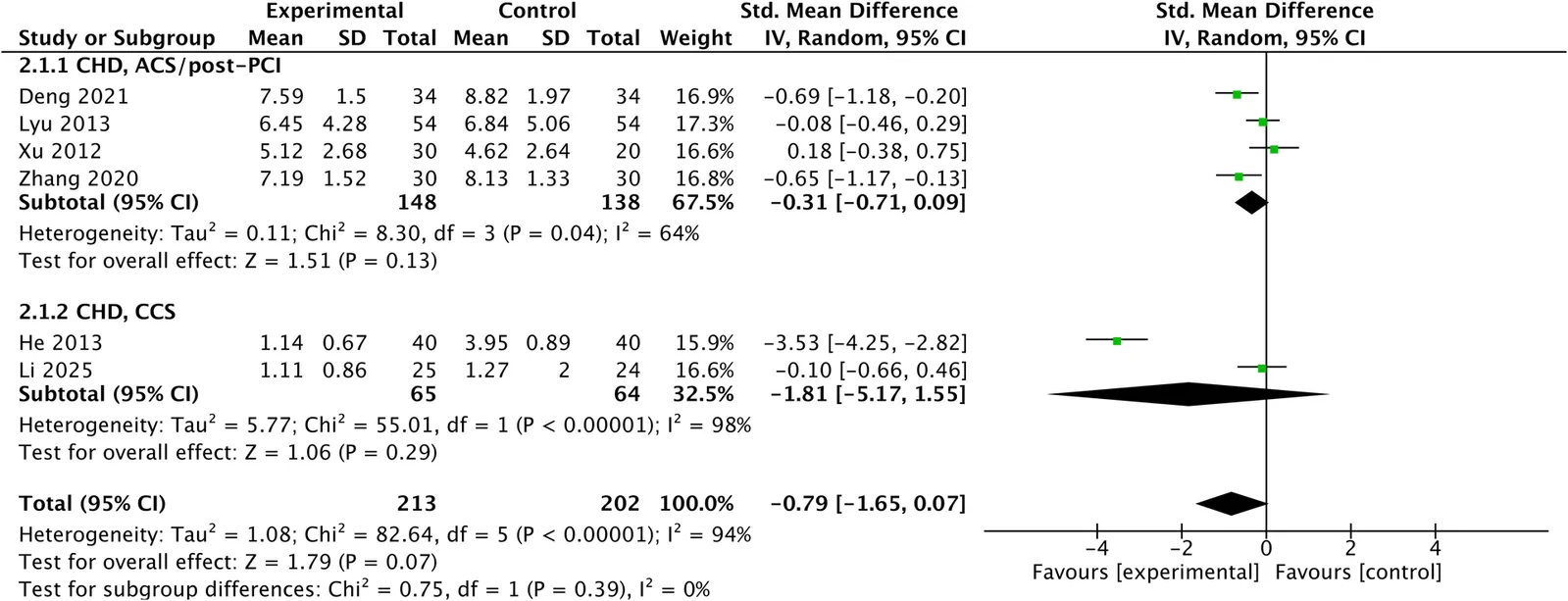
Subgroup forest plot for hs-CRP.

#### 3.4.2 CRP

Five studies reported CRP (28, 32–34, 36). Acupuncture interventions were associated with lower CRP levels (SMD = −0.87, 95% CI −1.43 to −0.30, *P* = 0.003; I^2^ = 85%; *τ*^2^ = 0.34). However, the 95% PI ranged from −2.15 to 0.42 and included no effect, indicating uncertainty regarding the effect expected in a future comparable study. The reduction was statistically significant in the ACS/PCI subgroup but not in the CHD with non-cardiac surgery subgroup; the test for subgroup interaction was not significant (*P* = 0.39; Figure 5). The certainty of evidence was low.

**Figure 5:**
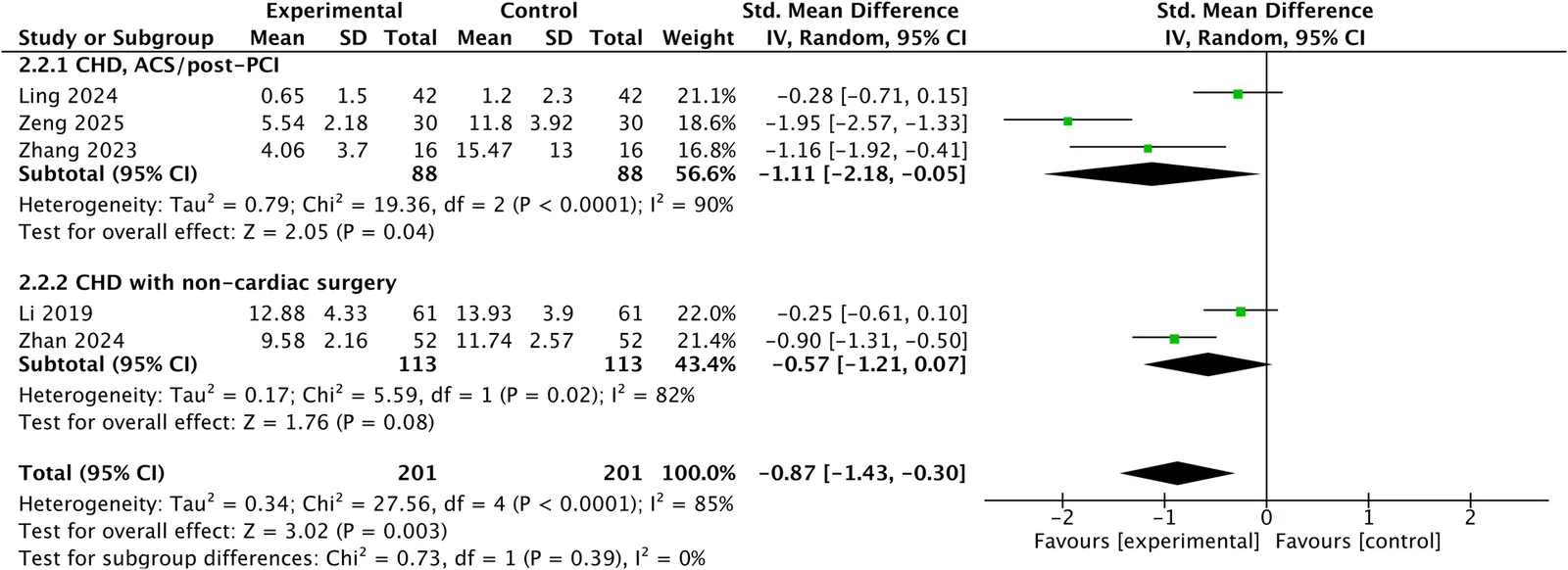
Subgroup forest plot for CRP.

#### 3.4.3 IL-6

Four studies reported IL-6 (29, 30, 33, 34). IL-6 levels were lower in the acupuncture-related intervention group (SMD = −0.74, 95% CI −1.31 to −0.17, *P* = 0.01; I^2^ = 84%; *τ*^2^ = 0.28). The 95% PI ranged from −1.93 to 0.45 and crossed the null. The effect was statistically significant in the CHD with non-cardiac surgery subgroup but not in the ACS/PCI subgroup, and the test for subgroup interaction was statistically significant (*P* = 0.04; Figure 6). Because only four studies contributed to this comparison, the interaction should be viewed as exploratory. The certainty of evidence was low.

**Figure 6:**
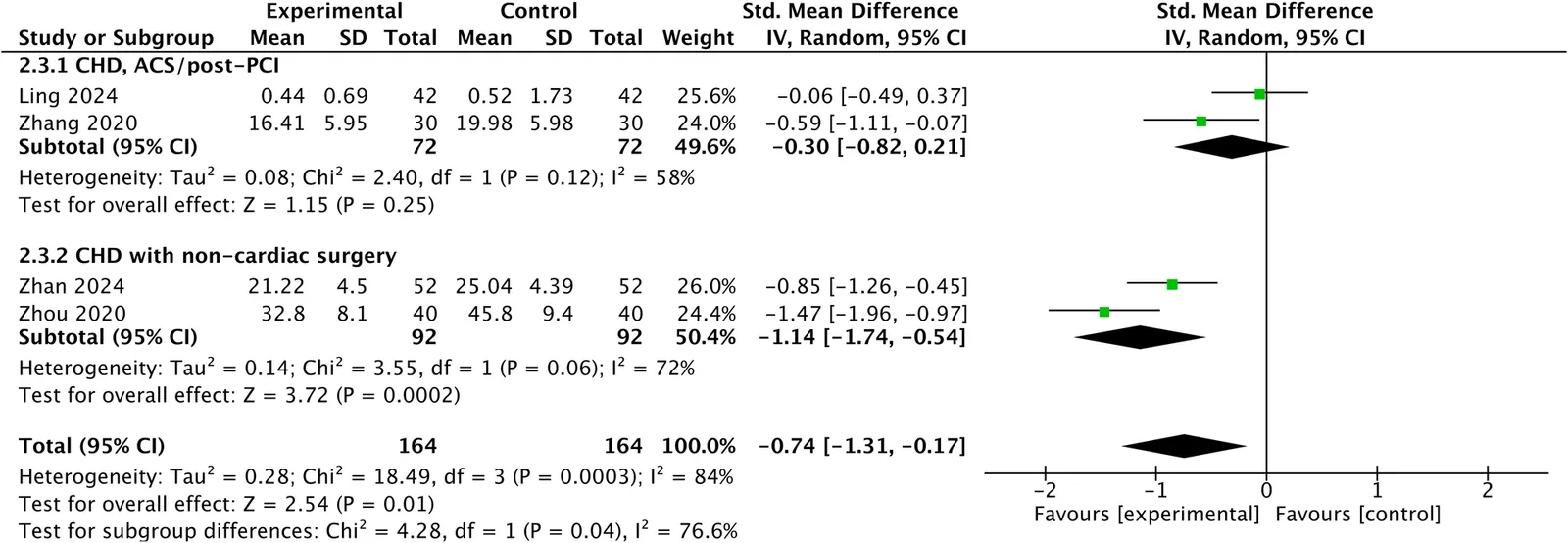
Subgroup forest plot for IL-6.

#### 3.4.4 TNF-α

Three studies reported TNF-α (30, 33, 34). The pooled estimate favored acupuncture interventions (SMD = −1.60, 95% CI −3.15 to −0.06, *P* = 0.04; I^2^ = 97%; *τ*^2^ = 1.78), but the 95% PI was very wide (−4.65 to 1.44) and crossed the null. The effect is therefore highly uncertain. The certainty of evidence was very low. The forest plot is shown in Figure 7.

**Figure 7:**
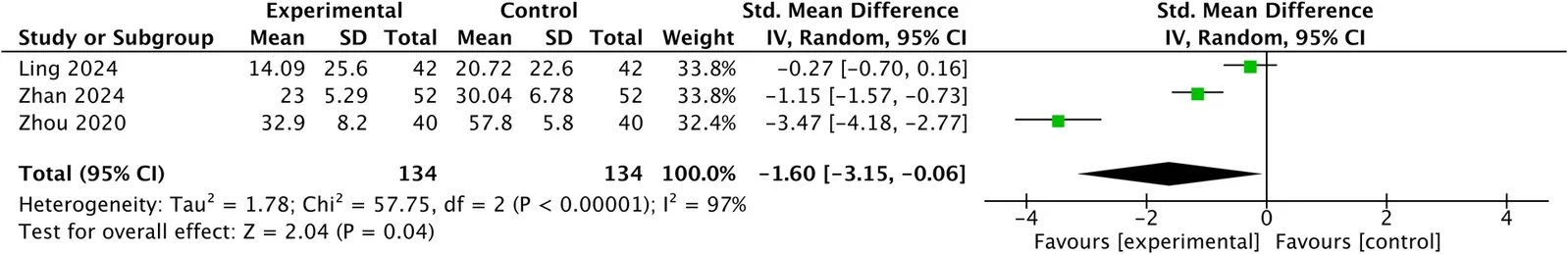
TNF-α forest plot.

#### 3.4.5 Other inflammation-related biomarkers

Several additional inflammation-related biomarkers were reported by individual studies and were summarized narratively. Wang et al. reported neutrophil count and NLR in patients with chronic stable angina. Compared with the medication group, the acupuncture group had a significantly lower NLR after treatment (*P* < 0.05), whereas neutrophil count did not differ significantly between groups (27).

Ling (34) reported IL-10 and IL-1β in patients after PCI for acute myocardial infarction (AMI). For IL-1β, the verum acupuncture group showed a significant decrease after treatment compared with baseline (*P* = 0.009), whereas no significant change was observed in the sham acupuncture group (*P* = 0.553). However, the between-group difference after treatment was not statistically significant (*P* = 0.295). For IL-10, no significant pre-post change was observed in the verum acupuncture group (*P* = 0.761), while the sham acupuncture group showed a significant decrease after treatment (*P* = 0.015). The between-group difference after treatment was also not statistically significant (*P* = 0.244). Zhan et al. (33) reported ICAM-1 and found that ICAM-1 levels at 1 and 3 days after surgery were lower in the acupuncture combined with intravenous anesthesia group than in the routine anesthesia group (*P* < 0.05) (33). Because NLR, neutrophil count, IL-10, IL-1β, and ICAM-1 were each reported by only one study, quantitative pooling was not performed; these findings were considered exploratory.

### 3.5 Lipid metabolism indicators

#### 3.5.1 TC

Four studies reported TC (24, 29, 31, 36). Acupuncture interventions significantly reduced TC compared with control interventions (MD = −0.42, 95% CI −0.58 to −0.26, *P* < 0.00001; I^2^ = 0%). The certainty of evidence was moderate. The forest plot is shown in Figure 8.

**Figure 8:**
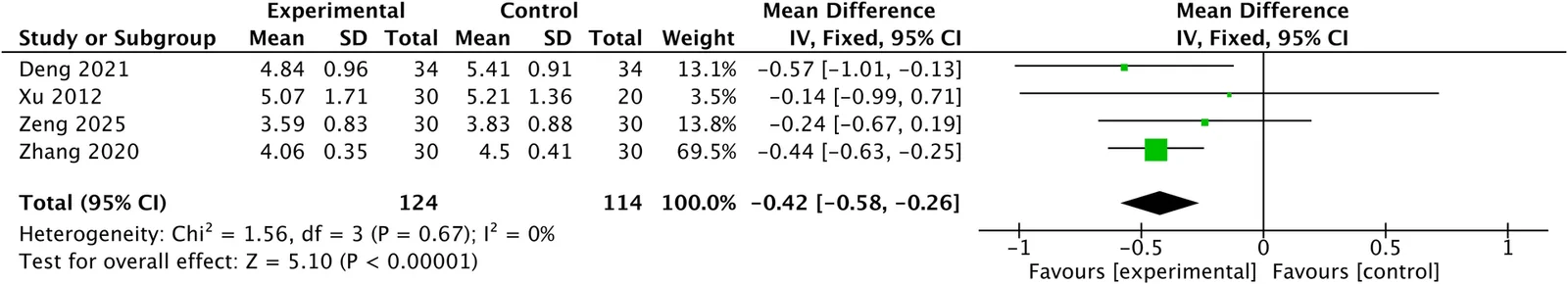
TC forest plot.

#### 3.5.2 TG

Four studies reported TG (24, 29, 31, 36). The pooled result showed a significant reduction in TG in the acupuncture-related intervention group (MD = −0.18, 95% CI −0.28 to −0.08, *P* = 0.0004; I^2^ = 16%). The certainty of evidence was moderate. The forest plot is shown in Figure 9.

**Figure 9:**
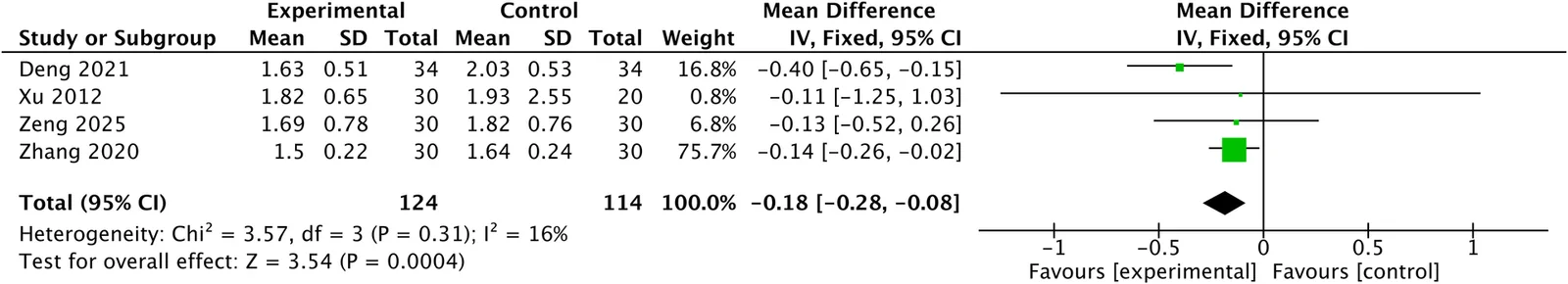
TG forest plot.

#### 3.5.3 LDL-C

Four studies reported LDL-C (24, 29, 31, 36). No significant difference was observed between groups (SMD = −0.13, 95% CI −0.55 to 0.29, *P* = 0.55; I^2^ = 62%). The certainty of evidence was very low. The forest plot is shown in Figure 10.

**Figure 10:**
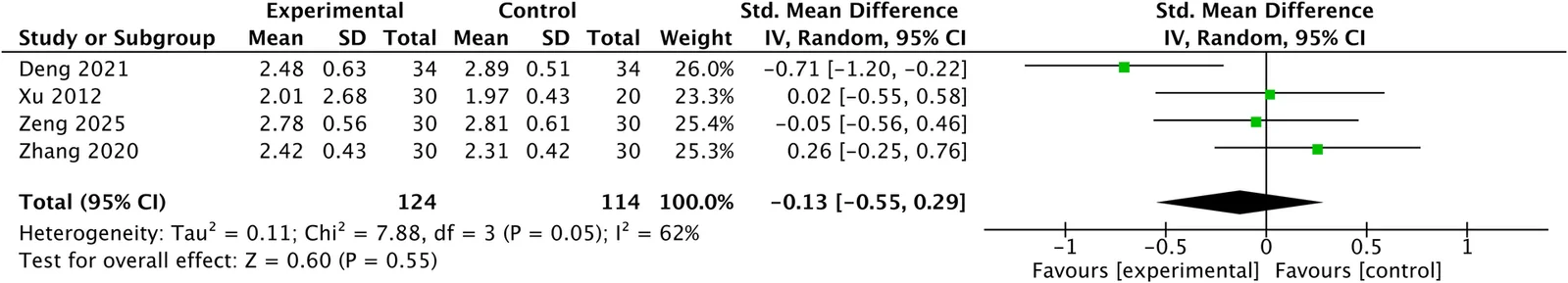
LDL-C forest plot.

#### 3.5.4 HDL-C

Four studies reported HDL-C (24, 29, 31, 36). The pooled result showed no significant difference between acupuncture interventions and control interventions (MD = 0.02, 95% CI −0.05 to 0.09, *P* = 0.54; I^2^ = 48%). The certainty of evidence was low. The forest plot is shown in Figure 11.

**Figure 11:**
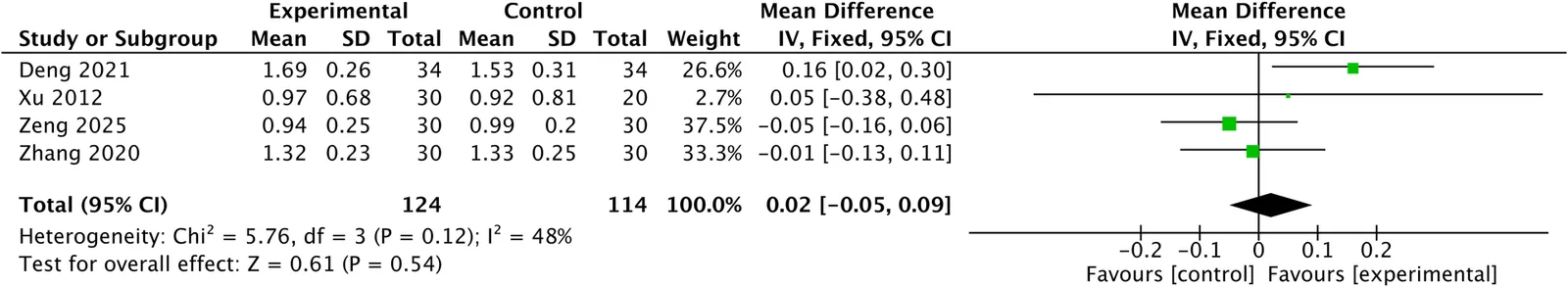
HDL-C forest plot.

### 3.6 ECG response

Three studies reported ECG response (25, 29, 36). The pooled result suggested a possible benefit of acupuncture interventions (RR = 2.39, 95% CI 1.00 to 5.72, *P* = 0.05; I^2^ = 89%). However, the confidence interval was wide, and the certainty of evidence was very low. The forest plot is shown in Figure 12.

**Figure 12:**
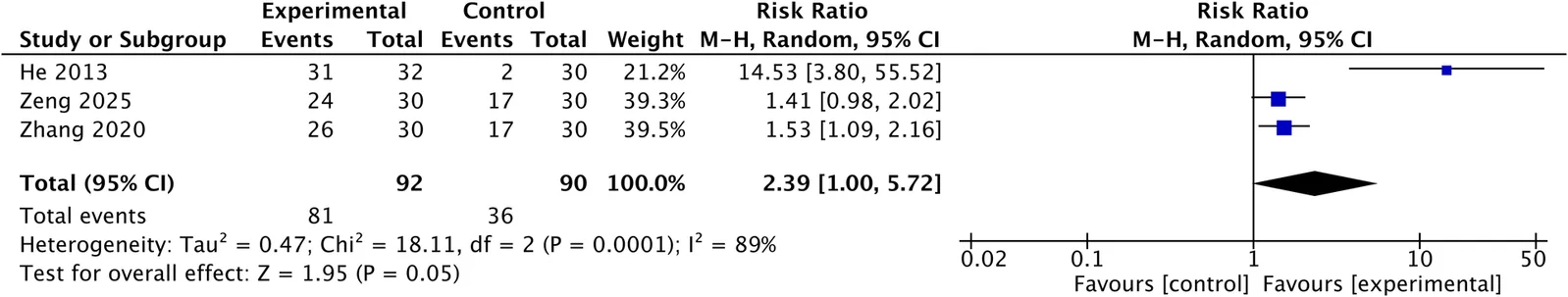
ECG response forest plot.

### 3.7 Cardiovascular events and adverse events

Six studies reported adverse events (24, 25, 32, 34–36). No significant difference was observed between acupuncture interventions and control interventions (RR = 0.88, 95% CI 0.41 to 1.85, *P* = 0.73; I^2^ = 44%). The forest plot is shown in Figure 13. The total number of events was small and reporting was not standardized; the certainty of evidence was therefore very low. Two studies reported MACE. Zhang et al. (32) reported 4/16 events in the acupuncture group and 12/16 in the drug-treatment group during follow-up to 8 weeks (*P* = 0.016). Its composite included death, malignant arrhythmia, heart failure, recurrent myocardial infarction, repeat PCI, and chest pain. Zhang (29) reported 2/30 events in the acupuncture group and 5/30 in the control group at 3 months after PCI (*P* = 0.228), based on myocardial infarction, arrhythmia, cardiac death, and recurrent angina. Because the event composites and follow-up schedules differed, the results were summarized narratively rather than pooled.

**Figure 13:**
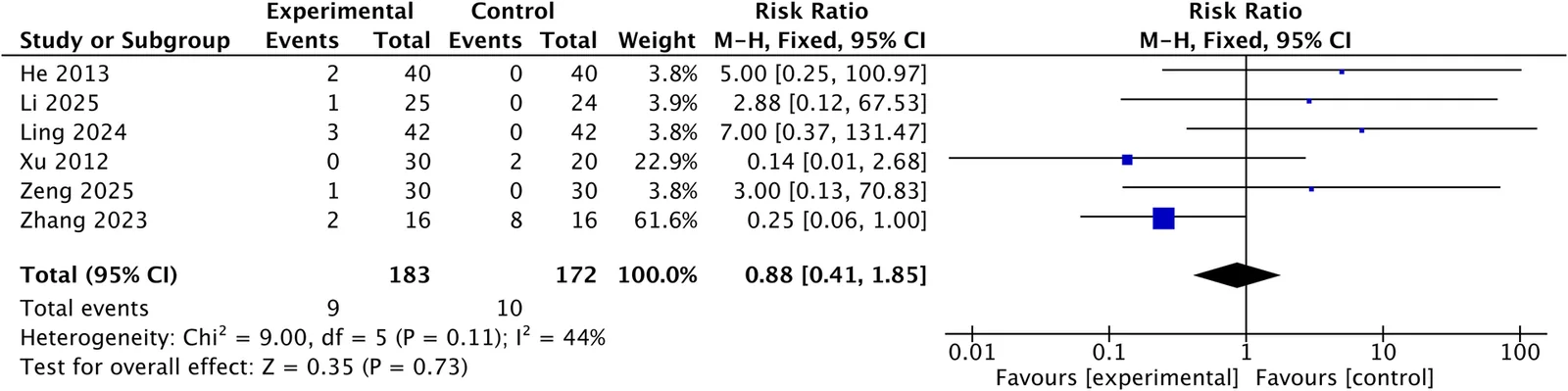
Adverse events forest plot.

### 3.8 Subgroup and sensitivity analyses

Subgroup analyses by clinical setting were performed for hs-CRP, CRP, and IL-6 (Figures 4–6). No interaction was observed for hs-CRP or CRP (both P for interaction = 0.39), whereas the interaction for IL-6 was statistically significant (*P* = 0.04). In the *post hoc* analysis by comparator type, the CRP estimate was smaller in sham-controlled trials (SMD = −0.26, 95% CI −0.54 to 0.01; I^2^ = 0%) than in non-sham-controlled trials (SMD = −1.32, 95% CI −1.98 to −0.66; I^2^ = 74%; P for interaction = 0.004). This interaction cannot be attributed solely to comparator design because comparator category coincided with differences in clinical setting and intervention characteristics across the five trials. An intervention-modality analysis was not undertaken after the five CRP studies were classified as three electroacupuncture studies, one TEAS study, and one eye-acupuncture study. Analyses by treatment duration and baseline inflammatory status were also not feasible. For hs-CRP, the available studies could not form comparable subgroups by intervention modality or comparator type. Detailed results are provided in Supplementary Table 6.

According to the prespecified rule, leave-one-out sensitivity analyses were performed only for outcomes with I^2^ ≥ 50%. For hs-CRP, the I^2^ values after sequential omission ranged from 54.0% to 95.2%; I^2^ decreased to 54.0% after omitting He 2013, suggesting that this study may be an important contributor to heterogeneity. For CRP, the I^2^ values ranged from 68.8% to 88.5%, and the overall direction of effect remained unchanged, indicating relatively stable findings. For IL-6 and TNF-α, the I^2^ values after sequential omission ranged from 68.1% to 89.1% and from 87.9% to 98.3%, respectively, suggesting persistent heterogeneity and limited robustness. For LDL-C, I^2^ decreased to 0% after omitting Deng 2021, indicating that this study may be the main source of heterogeneity. For ECG response, I^2^ decreased to 0% after omitting He 2013, suggesting that this study had a substantial influence on heterogeneity. Sensitivity analyses were not performed for TC, TG, HDL-C, or adverse events because their I^2^ values were below 50%. Details are presented in Supplementary Figures 1–6 and Supplementary Table 2.

### 3.9 Certainty of evidence

The certainty of evidence was moderate for TC and TG; low for CRP, IL-6, and HDL-C; and very low for hs-CRP, TNF-α, LDL-C, ECG response, and adverse events. Formal publication-bias tests were not undertaken because fewer than 10 studies contributed to each outcome. Qualitative assessment indicated that publication bias could not be ruled out because the evidence consisted mainly of small, single-center trials and selective outcome reporting remained possible. Detailed outcome-specific judgments are provided in Supplementary Tables 3 and 4.

## 4 Discussion

This systematic review and meta-analysis included 13 randomized trials involving 927 participants with CHD. The pooled estimates for CRP and IL-6 favored acupuncture-related interventions, but substantial heterogeneity and prediction intervals including no effect prevent these average estimates from being interpreted as a consistent anti-inflammatory effect across clinical settings. The hs-CRP estimate did not reach statistical significance, and the effect on TNF-α remained highly uncertain. Among secondary outcomes, TC and TG decreased, while LDL-C and HDL-C did not differ significantly. The evidence for ECG response, adverse events, and cardiovascular events was insufficient for firm conclusions.

CRP and IL-6 are closely linked components of the inflammatory cascade. IL-6 promotes hepatic synthesis of CRP and participates in endothelial activation and atherosclerotic inflammation; a parallel reduction in these markers is therefore biologically plausible. Inflammation plays a central role in atherosclerosis, plaque instability, and acute coronary events (6, 7), and randomized trials targeting inflammatory pathways have supported inflammation as a therapeutic target in selected patients with atherosclerotic cardiovascular disease (8–10). However, statistical significance of the pooled estimates should not be equated with a consistent treatment effect. The certainty of evidence for both outcomes was low, substantial between-study heterogeneity remained, and their prediction intervals included no effect. The observed reductions should therefore be regarded as preliminary evidence rather than confirmation of an anti-inflammatory effect.

The different findings for conventional CRP and hs-CRP warrant particular attention. These assays measure the same protein but are optimized for different concentration ranges. hs-CRP is primarily used to detect low-grade inflammation and residual inflammatory risk in cardiovascular research (37, 38), whereas conventional CRP is more responsive when inflammatory activity is more pronounced. The sets of studies contributing to the two analyses were not identical and covered different clinical contexts, from stable CHD to ACS, post-PCI states, and perioperative stress. Variation in disease phase, inflammatory burden, treatment exposure, and timing of assessment may therefore have contributed to the discrepant pooled estimates. Because the hs-CRP result was non-significant, highly heterogeneous, and supported by very-low-certainty evidence, the findings for conventional CRP should not be generalized to hs-CRP or interpreted as uniform suppression of cardiovascular inflammation.

Substantial heterogeneity was observed across the inflammatory outcomes. Clinical setting did not explain the variability in hs-CRP or CRP. The interaction observed for IL-6 may suggest a different response in patients undergoing non-cardiac surgery, in whom perioperative tissue injury and sympathetic activation can produce a marked but transient inflammatory response (39); however, this analysis involved only four studies and should therefore be interpreted as exploratory. The *post hoc* comparator analysis for CRP showed a near-null estimate in sham-controlled trials and a larger estimate in non-sham-controlled trials. This difference suggests that comparator design and treatment context may have contributed to the between-study variability. In particular, the larger effect observed in non-sham-controlled trials should not be interpreted as evidence of a specific biological anti-inflammatory effect of acupuncture, because such comparisons cannot fully distinguish acupuncture-specific effects from contextual effects, including expectation, attention, and co-interventions. Because comparator type also overlapped with differences in clinical setting and intervention characteristics, the observed interaction cannot be attributed to comparator type alone.

The prediction intervals provide a complementary perspective to the pooled confidence intervals. Although the confidence intervals for CRP and IL-6 excluded the null, the prediction intervals for hs-CRP, CRP, IL-6, and TNF-α all included no effect and extended into effects in the opposite direction. Thus, the average pooled estimates do not imply that a similar effect would be observed in every comparable clinical setting; a future study could report a smaller effect, no effect, or an effect in the opposite direction. This uncertainty is particularly relevant given the diversity of patient populations, treatment regimens, comparators, and clinical contexts.

The GRADE assessment further limits the strength of inference. Evidence was rated low for CRP and IL-6 and very low for hs-CRP and TNF-α. Risk-of-bias concerns arose mainly from incomplete reporting of allocation concealment, blinding, and prespecified analysis plans, while marked heterogeneity reduced confidence in the consistency of the effects. For some outcomes, sparse data and wide confidence intervals also limited precision. Accordingly, the current evidence is insufficient to define the magnitude of any anti-inflammatory effect or identify the patients most likely to benefit.

Clinical relevance cannot be inferred from biomarker changes alone. CRP, hs-CRP, IL-6, and TNF-α are associated with cardiovascular risk, but lowering a biomarker does not necessarily reduce myocardial infarction, cardiovascular death, or revascularization. Only two small trials reported short-term MACE, and their composite definitions and follow-up periods differed; one composite also included chest pain. These data were not suitable for pooling and do not establish an effect on cardiovascular prognosis. Acupuncture should therefore be considered, at most, an adjunctive intervention and not an alternative to guideline-directed treatment.

Previous reviews have suggested possible benefits of acupuncture for angina symptoms, electrocardiographic response, or glucose and lipid metabolism, although the certainty of that evidence has generally remained limited (14–18). The present review adds a focused synthesis of inflammatory biomarkers. Experimental studies indicate that acupuncture, particularly electroacupuncture, may modulate inflammation through neuroimmune and somatosensory-autonomic pathways (19, 20). These mechanisms provide biological context but do not resolve the uncertainty of the clinical evidence, because the included trials did not directly test the proposed pathways.

This review has several limitations. The evidence base was small for most outcomes, and all trials were conducted in China, limiting precision and generalizability. Most studies were small and single-center, and selective publication or outcome reporting cannot be ruled out. Differences in disease phase, intervention protocols, control conditions, and follow-up contributed to heterogeneity, while the available studies did not support robust analyses of several potential effect modifiers. Some subgroup analyses were undertaken *post hoc* and should be regarded as exploratory. Because few trials used credible sham controls, the current evidence cannot reliably separate acupuncture-specific effects from non-specific or contextual effects. Reporting of adverse events was incomplete and non-standardized. Finally, the evidence was dominated by surrogate biomarkers, with insufficient long-term cardiovascular outcomes to determine clinical benefit.

## 5 Conclusion

The pooled analyses yielded lower average CRP and IL-6 estimates in patients with CHD, but substantial heterogeneity, low-certainty evidence, and prediction intervals including no effect do not support a consistent or acupuncture-specific anti-inflammatory benefit. Current studies cannot separate specific acupuncture effects from non-specific or contextual effects. Findings for hs-CRP and TNF-α remained inconclusive, and evidence on cardiovascular outcomes and safety was limited. Future trials should use rigorous sham-controlled designs, consistent intervention and comparator protocols, standardized biomarker assessment, and longer clinically relevant follow-up.

## Data Availability

The original contributions presented in the study are included in the article/Supplementary Material, further inquiries can be directed to the corresponding authors.
