# Supplementary material for: Effects and safety of acupuncture on inflammatory biomarkers in patients with coronary heart disease: a systematic review and meta-analysis
Source: Front Cardiovasc Med. 2026 Oct 9;13:1891405. doi: 10.3389/fcvm.2026.1891405 (PMC13690532; doi:10.3389/fcvm.2026.1891405)
Supplement: Supplementary file 1 [file Datasheet2.docx]

Supplementary Material

# 1 Supplementary Database Search Terms

**Search strategy note:** All eight database searches were last run on 12 March 2026. Platforms were PubMed (National Library of Medicine), Embase (Elsevier), the Cochrane Library (Wiley), Web of Science Core Collection (Clarivate), CNKI, Wanfang Data, VIP, and SinoMed. The database-specific strategies below reproduce the controlled vocabulary, free-text terms, field identifiers, Boolean operators, and filters or limits used for each source. Proximity operators were not added retrospectively where they were not part of the executed strategy.

The terms were informed by previous related reviews and adapted to the indexing and syntax of each database. The strategies below reproduce the searches conducted on 12 March 2026. The selected databases include journal articles as well as indexed dissertations, conference records, and trial records where available.

PubMed (National Library of Medicine; searched 12 March 2026)

**#1** (Coronary Disease[Mesh]) OR (Coronary Disease[Title/Abstract]) OR (Coronary Artery Disease[Mesh]) OR (Coronary Artery Disease[Title/Abstract]) OR (Coronary Heart Disease[Title/Abstract]) OR (Acute coronary syndrome[Mesh]) OR (Acute coronary syndrome[Title/Abstract]) OR (Angina Pectoris[Mesh]) OR (Angina Pectoris[Title/Abstract]) OR (Myocardial Infarction[Mesh]) OR (Myocardial Infarct[Title/Abstract]) OR (Heart Attack[Title/Abstract]) OR (Cardiovascular Stroke[Title/Abstract]) OR (Chronic coronary syndrome[Title/Abstract]) OR (Ischemic heart disease[Title/Abstract])

**#2** (Acupuncture[Mesh]) OR (Acupuncture[Title/Abstract]) OR (Pharmacopuncture[Title/Abstract]) OR (Acupuncture Therapy[Mesh]) OR (Acupuncture Therapy[Title/Abstract]) OR (Acupuncture Treatment[Title/Abstract]) OR (Pharmacoacupuncture Treatment[Title/Abstract]) OR (Acupotomy[Title/Abstract]) OR (Acupuncture, Ear[Mesh]) OR (Auricular Acupuncture[Title/Abstract]) OR (Acupuncture Points[Mesh]) OR (Electroacupuncture[Title/Abstract]) OR (Needle Warming[Title/Abstract]) OR (body acupuncture[Title/Abstract]) OR (abdominal acupuncture[Title/Abstract]) OR (fire needle[Title/Abstract]) OR (Moxibustion[Mesh]) OR (Moxabustion[Title/Abstract]) OR (Transcutaneous electrical acupoint stimulation[Title/Abstract])

**#3** (Randomized controlled trial[Filter])

**#4 #1** AND **#2** AND **#3**

Web of Science Core Collection (Clarivate; searched 12 March 2026)

**#1** TS=((Coronary Disease) OR (Coronary Artery Disease) OR (Coronary Heart Disease) OR (Acute coronary syndrome) OR (Angina Pectoris) OR (Myocardial Infarction) OR (Heart Attack) OR (Cardiovascular Stroke) OR (Chronic coronary syndrome) OR (Ischemic heart disease))

**#2** TS=((Acupuncture) OR (Pharmacopuncture) OR (Acupuncture Therapy) OR (Acupuncture Treatment) OR (Pharmacoacupuncture Treatment) OR (Acupotomy) OR (Acupuncture, Ear) OR (Auricular Acupuncture) OR (Acupuncture Points) OR (Electroacupuncture) OR (Needle Warming) OR (body acupuncture) OR (abdominal acupuncture) OR (fire needle) OR (Moxibustion) OR (Moxabustion) OR (Transcutaneous electrical acupoint stimulation))

**#3** TS=("randomized controlled trial" OR "randomised controlled trial" OR "randomized clinical trial" OR "randomised clinical trial" OR RCT)

**#4 #1** AND **#2** AND **#3**

Embase (Elsevier; searched 12 March 2026)

**#1** 'coronary artery disease'/exp or 'coronary artery disease':ab,ti or 'multivessel coronary artery disease':ab,ti or 'coronary disease':ab,ti or 'chronic coronary syndrome':ab,ti or 'acute coronary syndrome':ab,ti or 'angina pectoris'/exp or 'angina':ab,ti or 'anginal attack':ab,ti or 'stenocardia':ab,ti or 'angina pectoris':ab,ti or 'heart infarction'/exp or 'cardiac infarct':ab,ti or 'cardiac infarction':ab,ti or 'cardial infarct':ab,ti or 'heart attack':ab,ti or 'heart infarct':ab,ti or 'heart micro infarction':ab,ti or 'heart muscle infarction':ab,ti or 'infarction, heart':ab,ti or 'myocardial infarct':ab,ti or 'myocardial infarction':ab,ti or 'myocardium infarct':ab,ti or 'myocardium infarction':ab,ti or 'premonitory infarction sign':ab,ti or 'second heart attack':ab,ti or 'subendocardial infarction':ab,ti or 'transmural cardiac infarction':ab,ti or 'transmural heart infarction':ab,ti or 'transmural infarction, heart':ab,ti or 'heart infarction':ab,ti or 'coronary heart disease':ab,ti

**#2** 'acupuncture'/exp or 'acupuncture':ab,ti or 'acupuncture therapy':ab,ti or 'shonishin':ab,ti or 'acupotomy (needle-knife) therapy':ab,ti or 'needle-knife therapy':ab,ti or 'small needle-knife therapy':ab,ti or 'acupotomy':ab,ti or 'acupuncture, ear':ab,ti or 'acupuncture, earlobe':ab,ti or 'auriculo-acupuncture':ab,ti or 'auriculoacupuncture':ab,ti or 'auriculotherapy':ab,ti or 'ear acupuncture':ab,ti or 'earlobe acupuncture':ab,ti or 'auricular acupuncture':ab,ti or 'acupuncture, electric':ab,ti or 'electric acupuncture':ab,ti or 'electrical acupoint stimulation':ab,ti or 'electrical acupuncture':ab,ti or 'electro-acupuncture':ab,ti or 'electrode acupuncture':ab,ti or 'electronic acupuncture':ab,ti or 'electroacupuncture':ab,ti or 'herb acupuncture':ab,ti or 'herbal acupuncture':ab,ti or 'herbalized acupuncture':ab,ti or 'pharmaco-acupuncture':ab,ti or 'pharmaco-puncture':ab,ti or 'pharmacoacupuncture':ab,ti or 'pharmacopuncture':ab,ti or 'burnt needle therapy':ab,ti or 'fire acupuncture':ab,ti or 'fire needle acupuncture':ab,ti or 'fire needle therapy':ab,ti or 'fire needling':ab,ti or 'heat acupuncture':ab,ti or 'heat-type acupuncture':ab,ti or 'thermal acupuncture':ab,ti or 'thermo-acupuncture':ab,ti or 'thermoacupuncture':ab,ti or 'warm needle acupuncture':ab,ti or 'warm acupuncture':ab,ti or 'acu-point':ab,ti or 'acupoint':ab,ti or 'acupoints':ab,ti or 'acupuncture point'/exp or 'acupuncture point':ab,ti or 'point, acupuncture':ab,ti or 'acupuncture point':ab,ti or 'moxibustion'/exp or 'moxibustion':ab,ti or 'body acupuncture':ab,ti or 'abdominal acupuncture':ab,ti

**#3** ('randomized controlled trial'/de OR 'randomized controlled trial topic'/de)

**#4 #1** AND **#2** AND **#3**

Cochrane Library (Wiley; searched 12 March 2026)

#1 MeSH descriptor: [Coronary Disease] explode all trees

#2 MeSH descriptor: [Myocardial Infarction] explode all trees

#3 MeSH descriptor: [Coronary Artery Disease] explode all trees

#4 MeSH descriptor: [Acute Coronary Syndrome] explode all trees

#5 MeSH descriptor: [Angina Pectoris] explode all trees

#6 (Coronary Disease):ti,ab,kw OR (Heart Disease, Coronary):ti,ab,kw OR (Diseases, Coronary Heart):ti,ab,kw OR (Diseases, Coronary):ti,ab,kw OR (Disease, Coronary Heart):ti,ab,kw OR (Coronary Heart Disease):ti,ab,kw OR (Coronary Artery Disease):ti,ab,kw OR (Arteriosclerosis, Coronary):ti,ab,kw OR (Arterioscleroses, Coronary):ti,ab,kw OR (Coronary Arteriosclerosis):ti,ab,kw OR (Coronary Atheroscleroses):ti,ab,kw OR (Coronary Atherosclerosis):ti,ab,kw OR (Atherosclerosis, Coronary):ti,ab,kw OR (Coronary Arterioscleroses):ti,ab,kw OR (Atheroscleroses, Coronary):ti,ab,kw OR (Left Main Disease):ti,ab,kw OR (Left Main Coronary Disease):ti,ab,kw OR (Left Main Diseases):ti,ab,kw OR (Left Main Coronary Artery Disease):ti,ab,kw OR (Artery Diseases, Coronary):ti,ab,kw OR (Artery Disease, Coronary):ti,ab,kw OR (Coronary Artery Diseases):ti,ab,kw OR (Acute Coronary Syndrome):ti,ab,kw OR (Coronary Syndromes, Acute):ti,ab,kw OR (Acute Coronary Syndromes):ti,ab,kw OR (Syndrome, Acute Coronary):ti,ab,kw OR (Syndromes, Acute Coronary):ti,ab,kw OR (Coronary Syndrome, Acute):ti,ab,kw OR (Angina Pectoris):ti,ab,kw OR (Angor Pectoris):ti,ab,kw OR (Stenocardias):ti,ab,kw OR (Stenocardia):ti,ab,kw OR (Myocardial Infarction):ti,ab,kw OR (Infarcts, Myocardial):ti,ab,kw OR (Heart Attacks):ti,ab,kw OR (Stroke, Cardiovascular):ti,ab,kw OR (Infarct, Myocardial):ti,ab,kw OR (Cardiovascular Strokes):ti,ab,kw OR (Infarction, Myocardial):ti,ab,kw OR (Myocardial Infarct):ti,ab,kw OR (Strokes, Cardiovascular):ti,ab,kw OR (Infarctions, Myocardial):ti,ab,kw OR (Myocardial Infarcts):ti,ab,kw OR (Myocardial Infarctions):ti,ab,kw OR (Heart Attack):ti,ab,kw OR (Cardiovascular Stroke):ti,ab,kw

**#7** **#1** OR **#2** OR **#3** OR **#4** OR **#5** OR **#6**

**#8** MeSH descriptor: [Acupuncture] explode all trees

**#9** MeSH descriptor: [Acupuncture Therapy] explode all trees

**#10** MeSH descriptor: [Moxibustion] explode all trees

**#11** MeSH descriptor: [Acupuncture, Ear] explode all trees

**#12** MeSH descriptor: [Acupuncture Points] explode all trees

**#13** MeSH descriptor: [Electroacupuncture] explode all trees

**#14** (Acupuncture):ti,ab,kw OR (Pharmacopuncture):ti,ab,kw OR (Moxibustion):ti,ab,kw OR (Moxabustion):ti,ab,kw OR (Acupuncture Therapy):ti,ab,kw OR (Acupotomy):ti,ab,kw OR (Acupotomies):ti,ab,kw OR (Treatment, Acupuncture):ti,ab,kw OR (Therapy, Acupuncture):ti,ab,kw OR (Acupuncture Treatment):ti,ab,kw OR (Acupuncture Treatments):ti,ab,kw OR (Therapy, Pharmacoacupuncture):ti,ab,kw OR (Pharmacoacupuncture Therapy):ti,ab,kw OR (Treatment, Pharmacoacupuncture):ti,ab,kw OR (Pharmacoacupuncture Treatment):ti,ab,kw OR (Acupuncture, Ear):ti,ab,kw OR (Acupunctures, Auricular):ti,ab,kw OR (Acupuncture, Auricular):ti,ab,kw OR (Auricular Acupuncture):ti,ab,kw OR (Auricular Acupunctures):ti,ab,kw OR (Ear Acupuncture):ti,ab,kw OR (Ear Acupunctures):ti,ab,kw OR (Acupunctures, Ear):ti,ab,kw OR (Acupuncture Points):ti,ab,kw OR (Acupoint):ti,ab,kw OR (Point, Acupuncture):ti,ab,kw OR (Acupuncture Point):ti,ab,kw OR (Points, Acupuncture):ti,ab,kw OR (Acupoints):ti,ab,kw OR (Electroacupuncture):ti,ab,kw OR (Needle Warming):ti,ab,kw OR (body acupuncture):ti,ab,kw OR (abdominal acupuncture):ti,ab,kw OR (fire needle):ti,ab,kw

**#15** **#8** OR **#9** OR **#10** OR **#11** OR **#12** OR **#13** OR **#14**

**#16** **#7** AND **#15**

CNKI (searched 12 March 2026)

(主题:冠状动脉疾病)OR(主题:冠心病)OR(主题:心肌梗死)OR(主题:心绞痛)OR(篇关摘:冠状动脉疾病+冠状动脉硬化+冠状动脉动脉粥样硬化 +冠状动脉粥样硬化+冠心病+冠状动脉心脏病 + 冠状动脉粥样硬化性心脏病 + 心肌梗死 + 心肌梗塞 +心脏病发作 +心绞痛 + 心绞疼 + 狭心症 +冠脉综合征)OR(主题:胸痹)OR(主题:真心痛)OR(篇关摘:胸痹 +胸痹心痛 + 真心痛)AND(主题:针灸疗法)OR(主题:针刺)OR(主题:针刺穴位)OR(主题:针刺，耳)OR(主题:电针)OR(主题:温针疗法)OR(主题:灸法)OR(篇关摘:针灸疗法+针灸+针刺+药物针刺+针刺穴位 + 穴位 + 耳针 + 耳针刺 + 耳廓针刺 + 电针 + 温针疗法 + 温针灸+ 温和灸 +灸法+艾灸)AND(主题:随机对照试验)OR(篇关摘:随机对照试验 + 随机对照研究 +随机临床试验 + 随机对照 + RCT)

Wanfang Data (searched 12 March 2026)

(主题:(冠状动脉疾病 or 冠心病 or 心肌梗死 or 心绞痛 or 胸痹 or 真心痛) or 题名或关键词:(冠状动脉疾病 or 冠状动脉硬化 or 冠状动脉动脉粥样硬化 or 冠状动脉粥样硬化 or 冠心病 or 冠状动脉心脏病 or 冠状动脉粥样硬化性心脏病 or 心肌梗死 or 心肌梗塞 or 心脏病发作 or 心绞痛 or 心绞疼 or 狭心症 or 冠脉综合征 or 胸痹 or 胸痹心痛 or 真心痛)) and (主题:(针灸疗法 or 针刺 or 针刺穴位 or 针刺，耳 or 电针 or 温针疗法 or 灸法) or 题名或关键词:(针灸疗法 or 针灸 or 针刺 or 药物针刺 or 针刺穴位 or 穴位 or 耳针 or 耳针刺 or 耳廓针刺 or 电针 or 温针疗法 or 温针灸 or 温和灸 or 灸法 or 艾灸)) and (主题:(随机对照试验) or 题名或关键词:(随机对照试验 or 随机对照研究 or 随机临床试验 or 随机对照 or RCT))

VIP Database (searched 12 March 2026)

(SU=冠状动脉疾病 OR SU=冠心病 OR SU=心肌梗死 OR SU=心绞痛 OR SU=胸痹 OR SU=真心痛OR M=冠状动脉疾病 OR M=冠状动脉硬化 OR M=冠状动脉动脉粥样硬化 OR M=冠状动脉粥样硬化OR M=冠心病 OR M=冠状动脉心脏病 OR M=冠状动脉粥样硬化性心脏病OR M=心肌梗死 OR M=心肌梗塞 OR M=心脏病发作 OR M=心绞痛 OR M=心绞疼 OR M=狭心症OR M=冠脉综合征 OR M=胸痹 OR M=胸痹心痛 OR M=真心痛OR R=冠状动脉疾病 OR R=冠状动脉硬化 OR R=冠状动脉动脉粥样硬化 OR R=冠状动脉粥样硬化OR R=冠心病 OR R=冠状动脉心脏病 OR R=冠状动脉粥样硬化性心脏病OR R=心肌梗死 OR R=心肌梗塞 OR R=心脏病发作 OR R=心绞痛 OR R=心绞疼 OR R=狭心症OR R=冠脉综合征 OR R=胸痹 OR R=胸痹心痛 OR R=真心痛) AND (SU=针灸疗法 OR SU=针刺 OR SU=针刺穴位 OR SU=针刺，耳 OR SU=电针 OR SU=温针疗法 OR SU=灸法OR M=针灸疗法 OR M=针灸 OR M=针刺 OR M=药物针刺 OR M=针刺穴位 OR M=穴位OR M=耳针 OR M=耳针刺 OR M=耳廓针刺 OR M=电针 OR M=温针疗法 OR M=温针灸OR M=温和灸 OR M=灸法 OR M=艾灸OR R=针灸疗法 OR R=针灸 OR R=针刺 OR R=药物针刺 OR R=针刺穴位 OR R=穴位OR R=耳针 OR R=耳针刺 OR R=耳廓针刺 OR R=电针 OR R=温针疗法 OR R=温针灸OR R=温和灸 OR R=灸法 OR R=艾灸) AND (SU=随机对照试验OR M=随机对照试验 OR M=随机对照研究 OR M=随机临床试验 OR M=随机对照 OR M=RCT

OR R=随机对照试验 OR R=随机对照研究 OR R=随机临床试验 OR R=随机对照 OR R=RCT)

SinoMed (searched 12 March 2026)

("随机对照试验"[常用字段:智能] OR "随机对照研究"[常用字段:智能] OR "随机临床试验"[常用字段:智能] OR "随机对照"[常用字段:智能] OR "RCT"[常用字段:智能]) AND ("针灸疗法"[常用字段:智能] OR "针刺"[常用字段:智能] OR "针刺穴位"[常用字段:智能] OR "针刺，耳"[常用字段:智能] OR "电针"[常用字段:智能] OR "温针疗法"[常用字段:智能] OR "灸法"[常用字段:智能] OR "药物针刺"[常用字段:智能] OR "穴位"[常用字段:智能]) AND ("冠状动脉疾病"[常用字段:智能] OR "冠心病"[常用字段:智能] OR "心肌梗死"[常用字段:智能] OR "心绞痛"[常用字段:智能] OR "胸痹"[常用字段:智能] OR "真心痛"[常用字段:智能] OR "冠状动脉粥样硬化性心脏病"[常用字段:智能] OR "冠脉综合征"[常用字段:智能] OR "胸痹心痛"[常用字段:智能])

# 2 Supplementary Tables

## Supplementary Table 1 PRISMA 2020 Checklist

| **Section and Topic** | **Item #** | **Checklist item** | **Location where item is reported** |
| --- | --- | --- | --- |
| **TITLE** | | |  |
| Title | 1 | Identify the report as a systematic review. | Title |
| **ABSTRACT** | | |  |
| Abstract | 2 | See the PRISMA 2020 for Abstracts checklist. | Abstract |
| **INTRODUCTION** | | |  |
| Rationale | 3 | Describe the rationale for the review in the context of existing knowledge. | Introduction |
| Objectives | 4 | Provide an explicit statement of the objective(s) or question(s) the review addresses. | Introduction |
| **METHODS** | | |  |
| Eligibility criteria | 5 | Specify the inclusion and exclusion criteria for the review and how studies were grouped for the syntheses. | Methods: Eligibility criteria |
| Information sources | 6 | Specify all databases, registers, websites, organisations, reference lists and other sources searched or consulted to identify studies. Specify the date when each source was last searched or consulted. | Methods: Search strategy |
| Search strategy | 7 | Present the full search strategies for all databases, registers and websites, including any filters and limits used. | Methods: Search strategy; Supplementary Database Search Terms |
| Selection process | 8 | Specify the methods used to decide whether a study met the inclusion criteria of the review, including how many reviewers screened each record and each report retrieved, whether they worked independently, and if applicable, details of automation tools used in the process. | Methods: Literature management and data extraction |
| Data collection process | 9 | Specify the methods used to collect data from reports, including how many reviewers collected data from each report, whether they worked independently, any processes for obtaining or confirming data from study investigators, and if applicable, details of automation tools used in the process. | Methods: Literature management and data extraction |
| Data items | 10a | List and define all outcomes for which data were sought. Specify whether all results that were compatible with each outcome domain in each study were sought (e.g. for all measures, time points, analyses), and if not, the methods used to decide which results to collect. | Methods: Outcomes; Literature management and data extraction |
|  | 10b | List and define all other variables for which data were sought (e.g. participant and intervention characteristics, funding sources). Describe any assumptions made about any missing or unclear information. | Methods: Literature management and data extraction |
| Study risk of bias assessment | 11 | Specify the methods used to assess risk of bias in the included studies, including details of the tool(s) used, how many reviewers assessed each study and whether they worked independently, and if applicable, details of automation tools used in the process. | Methods: Risk of bias assessment |
| Effect measures | 12 | Specify for each outcome the effect measure(s) (e.g. risk ratio, mean difference) used in the synthesis or presentation of results. | Methods: Data synthesis and statistical analysis |
| Synthesis methods | 13a | Describe the processes used to decide which studies were eligible for each synthesis (e.g. tabulating the study intervention characteristics and comparing against the planned groups for each synthesis (item #5)). | Methods: Data synthesis and statistical analysis |
|  | 13b | Describe any methods required to prepare the data for presentation or synthesis, such as handling of missing summary statistics, or data conversions. | Methods: Data synthesis and statistical analysis |
|  | 13c | Describe any methods used to tabulate or visually display results of individual studies and syntheses. | Methods: Data synthesis and statistical analysis; Results |
|  | 13d | Describe any methods used to synthesize results and provide a rationale for the choice(s). If meta-analysis was performed, describe the model(s), method(s) to identify the presence and extent of statistical heterogeneity, and software package(s) used. | Methods: Data synthesis and statistical analysis |
|  | 13e | Describe any methods used to explore possible causes of heterogeneity among study results (e.g. subgroup analysis, meta-regression). | Methods: Data synthesis and statistical analysis; Results: Subgroup and sensitivity analyses; Supplementary Table 6 |
|  | 13f | Describe any sensitivity analyses conducted to assess robustness of the synthesized results. | Results: Subgroup and sensitivity analyses; Supplementary Table 2 |
| Reporting bias assessment | 14 | Describe any methods used to assess risk of bias due to missing results in a synthesis (arising from reporting biases). | Methods: Certainty of evidence |
| Certainty assessment | 15 | Describe any methods used to assess certainty (or confidence) in the body of evidence for an outcome. | Methods: Certainty of evidence |
| **RESULTS** | | |  |
| Study selection | 16a | Describe the results of the search and selection process, from the number of records identified in the search to the number of studies included in the review, ideally using a flow diagram. | Results: Study selection; Figure 1 |
|  | 16b | Cite studies that might appear to meet the inclusion criteria, but which were excluded, and explain why they were excluded. | Results: Study selection |
| Study characteristics | 17 | Cite each included study and present its characteristics. | Results: Characteristics of included studies; Table 1 |
| Risk of bias in studies | 18 | Present assessments of risk of bias for each included study. | Results: Risk of bias; Figures 2 and 3 |
| Results of individual studies | 19 | For all outcomes, present, for each study: (a) summary statistics for each group (where appropriate) and (b) an effect estimate and its precision (e.g. confidence/credible interval), ideally using structured tables or plots. | Results; Figures 4-13 |
| Results of syntheses | 20a | For each synthesis, briefly summarise the characteristics and risk of bias among contributing studies. | Results |
|  | 20b | Present results of all statistical syntheses conducted. If meta-analysis was done, present for each the summary estimate and its precision (e.g. confidence/credible interval) and measures of statistical heterogeneity. If comparing groups, describe the direction of the effect. | Results; Figures 4-13 |
|  | 20c | Present results of all investigations of possible causes of heterogeneity among study results. | Results: Subgroup and sensitivity analyses; Supplementary Table 6 |
|  | 20d | Present results of all sensitivity analyses conducted to assess the robustness of the synthesized results. | Results: Subgroup and sensitivity analyses; Supplementary Table 2 |
| Reporting biases | 21 | Present assessments of risk of bias due to missing results (arising from reporting biases) for each synthesis assessed. | Results: Certainty of evidence; Supplementary Table 4 |
| Certainty of evidence | 22 | Present assessments of certainty (or confidence) in the body of evidence for each outcome assessed. | Results: Certainty of evidence; Supplementary Tables 3 and 4 |
| **DISCUSSION** | | |  |
| Discussion | 23a | Provide a general interpretation of the results in the context of other evidence. | Discussion |
|  | 23b | Discuss any limitations of the evidence included in the review. | Discussion |
|  | 23c | Discuss any limitations of the review processes used. | Discussion |
|  | 23d | Discuss implications of the results for practice, policy, and future research. | Discussion; Conclusion |
| **OTHER INFORMATION** | | |  |
| Registration and protocol | 24a | Provide registration information for the review, including register name and registration number, or state that the review was not registered. | Abstract; Methods: Study registration and reporting |
|  | 24b | Indicate where the review protocol can be accessed, or state that a protocol was not prepared. | Methods: Study registration and reporting |
|  | 24c | Describe and explain any amendments to information provided at registration or in the protocol. | Methods: Study registration and reporting; Data synthesis and statistical analysis |
| Support | 25 | Describe sources of financial or non-financial support for the review, and the role of the funders or sponsors in the review. | Funding |
| Competing interests | 26 | Declare any competing interests of review authors. | Conflict of interest |
| Availability of data, code and other materials | 27 | Report which of the following are publicly available and where they can be found: template data collection forms; data extracted from included studies; data used for all analyses; analytic code; any other materials used in the review. | Data availability statement; Supplementary material |

*From:*  Page MJ, McKenzie JE, Bossuyt PM, Boutron I, Hoffmann TC, Mulrow CD, et al. The PRISMA 2020 statement: an updated guideline for reporting systematic reviews. BMJ 2021;372:n71. doi: 10.1136/bmj.n71

For more information, visit: <http://www.prisma-statement.org/>

**Supplementary Table 2** Leave-one-out sensitivity analyses.

| **Outcome** | **Omitted study** | **Effect estimate** | **95% CI** | **P value** | **I² (%)** |
| --- | --- | --- | --- | --- | --- |
| hs-CRP | Deng 2021 | SMD = −0.82 | −1.89 to 0.26 | 0.138 | 95.2 |
| hs-CRP | Lyu 2013 | SMD = −0.94 | −2.03 to 0.14 | 0.089 | 94.7 |
| hs-CRP | Xu 2012 | SMD = −0.99 | −1.99 to 0.01 | 0.053 | 94.8 |
| hs-CRP | Zhang 2020 | SMD = −0.82 | −1.89 to 0.24 | 0.129 | 95.2 |
| hs-CRP | He 2013 | SMD = −0.27 | −0.60 to 0.05 | 0.102 | 54.0 |
| hs-CRP | Li 2025 | SMD = −0.93 | −1.96 to 0.10 | 0.077 | 95.1 |
| CRP | Ling 2024 | SMD = −1.03 | −1.73 to −0.34 | 0.004 | 87.2 |
| CRP | Zeng 2025 | SMD = −0.59 | −1.00 to −0.18 | 0.005 | 68.8 |
| CRP | Zhang 2023 | SMD = −0.81 | −1.46 to −0.17 | 0.014 | 88.5 |
| CRP | Li 2019 | SMD = −1.04 | −1.71 to −0.37 | 0.002 | 84.6 |
| CRP | Zhan 2024 | SMD = −0.87 | −1.62 to −0.12 | 0.022 | 88.5 |
| IL-6 | Ling 2024 | SMD = −0.97 | −1.45 to −0.49 | <0.001 | 68.1 |
| IL-6 | Zhang 2020 | SMD = −0.79 | −1.55 to −0.02 | 0.045 | 89.1 |
| IL-6 | Zhan 2024 | SMD = −0.70 | −1.53 to 0.13 | 0.097 | 88.8 |
| IL-6 | Zhou 2020 | SMD = −0.50 | −0.99 to −0.01 | 0.044 | 72.0 |
| TNF-α | Ling 2024 | SMD = −2.29 | −4.57 to −0.02 | 0.048 | 96.8 |
| TNF-α | Zhan 2024 | SMD = −1.86 | −5.00 to 1.28 | 0.245 | 98.3 |
| TNF-α | Zhou 2020 | SMD = −0.71 | −1.57 to 0.15 | 0.104 | 87.9 |
| LDL-C | Deng 2021 | SMD = 0.08 | −0.22 to 0.38 | 0.614 | 0.0 |
| LDL-C | Xu 2012 | SMD = −0.17 | −0.73 to 0.39 | 0.551 | 73.4 |
| LDL-C | Zeng 2025 | SMD = −0.15 | −0.74 to 0.44 | 0.617 | 74.1 |
| LDL-C | Zhang 2020 | SMD = −0.26 | −0.72 to 0.21 | 0.274 | 58.1 |
| ECG response | He 2013 | RR = 1.47 | 1.15 to 1.89 | 0.002 | 0.0 |
| ECG response | Zeng 2025 | RR = 4.28 | 0.47 to 38.54 | 0.195 | 90.2 |
| ECG response | Zhang 2020 | RR = 4.13 | 0.42 to 40.25 | 0.223 | 90.8 |

CI, confidence interval; SMD, standardized mean difference; RR, risk ratio. Sensitivity analyses were conducted by sequentially omitting one study at a time for outcomes with substantial heterogeneity (I² ≥ 50%).

**Supplementary Table 3** Certainty of evidence assessed by GRADE.

| **Certainty assessment** | | | | | | | | **№ of patients** | | **Effect** | | **Certainty** |
| --- | --- | --- | --- | --- | --- | --- | --- | --- | --- | --- | --- | --- |
| **Outcome indicators** | **№ of studies** | **Study design** | **Risk of bias** | **Inconsistency** | **Indirectness** | **Imprecision** | **Other considerations** | **EG** | **CG** | **Relative (95% CI)** | **Absolute (95% CI)** |  |
| **hs-CRP** | 6 | randomised trials | serious | very serious | not serious | serious | Publication bias not downgraded* | 213 | 202 | - | SMD **0.79 lower** (1.65 lower to 0.07 higher) | ⨁◯◯◯ Very low |
| **CRP** | 5 | randomised trials | serious | serious | not serious | not serious | Publication bias not downgraded* | 201 | 201 | - | SMD **0.87 lower** (1.43 lower to 0.3 lower) | ⨁⨁◯◯ Low |
| **IL-6** | 4 | randomised trials | serious | serious | not serious | not serious | Publication bias not downgraded* | 164 | 164 | - | SMD **0.74 lower** (1.31 lower to 0.17 lower) | ⨁⨁◯◯ Low |
| **TNF-α** | 3 | randomised trials | serious | very serious | not serious | serious | Publication bias not downgraded* | 134 | 134 | - | SMD **1.6 lower** (3.15 lower to 0.06 lower) | ⨁◯◯◯ Very low |
| **LDL-C** | 4 | randomised trials | serious | serious | not serious | serious | Publication bias not downgraded* | 124 | 114 | - | SMD **0.13 lower** (0.55 lower to 0.29 higher) | ⨁◯◯◯ Very low |
| **TG** | 4 | randomised trials | serious | not serious | not serious | not serious | Publication bias not downgraded* | 124 | 114 | - | MD **0.18 lower** (0.28 lower to 0.08 lower) | ⨁⨁⨁◯ Moderate |
| **TC** | 4 | randomised trials | serious | not serious | not serious | not serious | Publication bias not downgraded* | 124 | 114 | - | MD **0.42 lower** (0.58 lower to 0.26 lower) | ⨁⨁⨁◯ Moderate |
| **HDL-C** | 4 | randomised trials | serious | not serious | not serious | serious | Publication bias not downgraded* | 124 | 114 | - | MD **0.02 higher** (0.05 lower to 0.09 higher) | ⨁⨁◯◯ Low |
| **ECG response** | 3 | randomised trials | serious | serious | serious | serious | Publication bias not downgraded* | 81/92 (88.0%) | 36/90 (40.0%) | RR 2.39 (1.00 to 5.72) | **556 more per 1,000** (from 0 fewer to 1,000 more) | ⨁◯◯◯ Very low |
| **AEs** | 6 | randomised trials | serious | not serious | serious | very serious | Publication bias not downgraded* | 9/183 (4.9%) | 10/172 (5.8%) | RR 0.88 (0.41 to 1.85) | **7 fewer per 1,000** (from 34 fewer to 49 more) | ⨁◯◯◯ Very low |

EG, experimental group; CG, control group; CI, confidence interval; MD, mean difference; RR, risk ratio; SMD, standardized mean difference.

*Formal publication-bias tests were not performed because each outcome included fewer than 10 studies. Publication bias was assessed qualitatively and could not be excluded; outcome-specific judgments are presented in Supplementary Table 4.

**Supplementary Table 4** Itemized GRADE domain judgments and downgrading rationales.

| **Outcome** | **Risk of bias** | **Inconsistency** | **Indirectness** | **Imprecision** | **Publication bias** | **Certainty** |
| --- | --- | --- | --- | --- | --- | --- |
| hs-CRP | Serious: allocation concealment/blinding/prespecified analyses insufficient in most trials. | Very serious: I²=94%, τ²=1.08, poor CI overlap, PI −2.99 to 1.42. | Not serious for the biomarker outcome. | Serious: CI crossed null; six small studies. | Not downgraded: six small, single-center studies; selective publication or outcome reporting cannot be ruled out. | Very low |
| CRP | Serious. | Serious: I²=85%, τ²=0.34; PI −2.15 to 0.42. | Not serious for the biomarker outcome. | Not serious: the pooled CI excluded the null and 402 participants contributed. | Not downgraded: five small, single-center studies from journals and theses; selective publication or outcome reporting cannot be ruled out. | Low |
| IL-6 | Serious: allocation concealment, blinding, or prespecified analyses were insufficiently reported in several trials. | Serious: I²=84%, τ²=0.28; the PI ranged from −1.93 to 0.45. | Not serious for the biomarker outcome. | Not serious: the pooled CI excluded the null. | Not downgraded: four small, single-center studies; selective publication or outcome reporting cannot be ruled out. | Low |
| TNF-α | Serious. | Very serious: I²=97%, τ²=1.78; markedly discordant effects. | Not serious for the biomarker outcome. | Serious: three studies; wide CI and PI −4.65 to 1.44. | Not downgraded: three small, single-center studies; selective publication or outcome reporting cannot be ruled out. | Very low |
| TC | Serious. | Not serious: I²=0%. | Not serious for the lipid outcome. | Not serious: CI was narrow and excluded null. | Not downgraded: four small studies; selective publication or outcome reporting cannot be ruled out. | Moderate |
| TG | Serious. | Not serious: I²=16%. | Not serious for the lipid outcome. | Not serious: CI excluded null. | Not downgraded: four small studies; selective publication or outcome reporting cannot be ruled out. | Moderate |
| LDL-C | Serious. | Serious: I²=62%, τ²=0.11; PI −0.92 to 0.66. | Not serious for the lipid outcome. | Serious: CI crossed null and included benefit and harm. | Not downgraded: four small studies; selective publication or outcome reporting cannot be ruled out. | Very low |
| HDL-C | Serious. | Not serious: I²=48%. | Not serious for the lipid outcome. | Serious: CI crossed null and effect was small. | Not downgraded: four small studies; selective publication or outcome reporting cannot be ruled out. | Low |
| ECG response | Serious. | Serious: I²=89%; one study strongly influenced the result. | Serious: non-standardized surrogate response definitions. | Serious: three studies and wide CI. | Not downgraded: three small studies with non-standardized response definitions; selective reporting cannot be ruled out. | Very low |
| Adverse events | Serious: incomplete and non-standardized ascertainment. | Not serious statistically (I²=44%), but event definitions varied. | Serious: sparse reporting did not capture the full safety domain. | Serious: few events and wide CI. | Not downgraded separately; selective non-reporting or under-reporting of harms cannot be ruled out. | Very low |

Note on publication bias: No outcome included 10 or more studies, so funnel plots and regression-based tests were not used. Publication bias was assessed qualitatively for each outcome from the number and size of studies, publication type, study setting, and potential selective outcome reporting. Publication bias was not assumed to be absent; the outcome-specific rationale for not downgrading is provided in Supplementary Table 4.

Note on imprecision: No validated minimally important difference was available for the standardized biomarker outcomes. A formal optimal information size threshold based on an assumed minimally important difference was therefore not applied. The available information size and pooled confidence interval were considered together. Prediction intervals were considered under inconsistency and were not used again as the sole reason to downgrade imprecision.

Note: Single-study outcomes (NLR, neutrophil count, IL-10, IL-1β, and ICAM-1) were not assigned a pooled GRADE rating because they did not constitute a quantitatively synthesized body of evidence.

**Supplementary Table 5** Prediction intervals for heterogeneous inflammatory outcomes.

| **Outcome** | **Studies** | **Pooled SMD (95% CI)** | **τ²** | **I²** | **95% prediction interval** |
| --- | --- | --- | --- | --- | --- |
| hs-CRP | 6 | −0.79 (−1.65 to 0.07) | 1.08 | 94% | −2.99 to 1.42 |
| CRP | 5 | −0.87 (−1.43 to −0.30) | 0.34 | 85% | −2.15 to 0.42 |
| IL-6 | 4 | −0.74 (−1.31 to −0.17) | 0.28 | 84% | −1.93 to 0.45 |
| TNF-α | 3 | −1.60 (−3.15 to −0.06) | 1.78 | 97% | −4.65 to 1.44 |

Prediction intervals were calculated under the random-effects model using a normal approximation. All intervals crossed the line of no effect and should be interpreted cautiously because few studies were available.

**Supplementary Table 6** Exploratory subgroup analyses and feasibility assessment.

| **Outcome** | **Potential effect modifier** | **Status** | **Result or reason** |
| --- | --- | --- | --- |
| hs-CRP | Clinical setting | Performed | No subgroup interaction (P=0.39). |
| hs-CRP | Intervention modality | Not feasible | Manual acupuncture: 4 studies; electroacupuncture: 1; TEAS: 1. |
| hs-CRP | Comparator type | Not feasible | No sham-controlled hs-CRP comparison was included in the quantitative synthesis; the three-arm TEAS trial contributed the TEAS plus conventional treatment versus conventional-treatment comparison. |
| hs-CRP | Duration ≥8 vs <8 weeks | Not feasible | Only one study used a course ≥8 weeks. |
| CRP | Clinical setting | Performed | No subgroup interaction (P=0.39). |
| CRP | Intervention modality | Not feasible | Electroacupuncture: 3 studies; TEAS: 1; eye acupuncture: 1. No modality comparison had at least two studies in each subgroup. |
| CRP | Comparator type | Post hoc exploratory | Sham-controlled SMD −0.26 (−0.54 to 0.01), I²=0%; non-sham-controlled SMD −1.32 (−1.98 to −0.66), I²=74%; interaction P=0.004. |
| CRP | Duration ≥8 vs <8 weeks | Not feasible | No CRP study used a course ≥8 weeks. |
| Inflammatory outcomes | Baseline inflammatory status | Not feasible | Baseline values were available in several trials, but the biomarkers, assays, clinical settings, units, and assessment schedules were not sufficiently comparable for subgroup analysis or meta-regression; only 3-6 studies contributed to each outcome. |

All analyses not specified in the original review plan are labelled post hoc and should be interpreted as hypothesis-generating.

**Supplementary Table 7** Additional details on background treatment, acupuncture and comparator procedures, biomarker assessment, and clinical endpoints.

| **Study** | **Background treatment/control** | **Acupuncture and comparator procedures** | **Biomarker assessment and MACE** |
| --- | --- | --- | --- |
| Xu (24) | Both groups received conventional ACS therapy, including antiplatelet, anticoagulation, coronary vasodilator, and beta-blocker treatment. The control group received atorvastatin nightly for 30 days; the dose was not reported. | EA at ST40, once daily, 30 min/session for 30 days; no sham procedure. | hs-CRP and lipids were assessed before and after 30 days. No MACE reported. |
| He (25) | Blank-control design; all groups received the same hospital meals. Concomitant cardiovascular medication was not reported. | Acupuncture at SP4, PC6, GB41, TE5, LU7, KI6, SI3, and BL62, once daily, 30 min/session for 2 weeks; the blank-control group received no acupuncture. | hs-CRP was measured before and after 2 weeks using a double-antibody sandwich ELISA. No MACE reported. |
| Lyu (26) | Both groups received conventional ACS treatment, described as antiplatelet, plaque-stabilizing, anticoagulation, and coronary vasodilator therapy; individual drugs and doses were not reported. | Acupuncture at PC6, HT7, HT6, and the chest-pain point, once daily, 30 min/session for 1 week; no sham procedure. | hs-CRP was assessed before and after the 1-week treatment course. No MACE reported. |
| Wang et al. (27) | Both groups received guideline-based basic medication and health education. Nitroglycerin 0.5 mg was used sublingually as needed; other drug details were not reported. | EA at PC6, once every other day, 30 min/session for 4 weeks; medication-only control. | Neutrophils and NLR were assessed at enrolment and day 30. No MACE reported. |
| Li et al. (28) | Both groups underwent the same standardized general anaesthesia. | TEAS at PC6 and PC4 in one perioperative session, from 30 min before induction to the end of surgery. Identical electrodes were placed without electrical stimulation in the sham group; participants were told that stimulation might or might not be felt. | CRP was assessed before anaesthesia induction and on postoperative days 1, 3, and 5. No MACE reported. |
| Zhang (29) | Both groups received aspirin 100 mg/day, clopidogrel 75 mg/day, atorvastatin 20 mg nightly, and metoprolol succinate extended release (47.5 mg, adjusted according to clinical status), with management of other risk factors. | Acupuncture at LU7, LI4, HT7, PC6, LI11, and LU5, five times/week, 45 min/session for 4 weeks; no sham procedure. | hs-CRP and IL-6 were assessed before and after 1 month. At 3 months, MACE comprised myocardial infarction, arrhythmia, cardiac death, and recurrent angina (2/30 vs 5/30). |
| Zhou et al. (30) | Both groups received standardized total intravenous anaesthesia; the control group received postoperative sufentanil patient-controlled intravenous analgesia. | EA at Neima point and PC6 in two perioperative sessions, 30 min/session; no sham procedure. | IL-6 and TNF-α were assessed before acupuncture and at 2, 12, 24, and 48 h after surgery. No MACE reported. |
| Deng et al. (31) | Both groups received isosorbide mononitrate 40 mg/day, aspirin 100 mg/day, and atorvastatin 20 mg nightly; nitroglycerin 0.5 mg was used sublingually as needed. | Acupuncture at CV17, BL15, PC6, ST36, and SP6, once daily, 30 min/session for 4 weeks; no sham procedure. | hs-CRP and lipids were assessed before and after 4 weeks. No MACE reported. |
| Zhang et al. (32) | Both groups received dual antiplatelet therapy after PCI (aspirin 100 mg/day and clopidogrel 75 mg/day for 2 weeks, with loading doses before PCI); other treatment was provided as clinically indicated. | Eye acupuncture at ACU1 and ACU2, once daily for 2 weeks, retained during 60-min exercise and removed after 5-min observation; drug-treatment control without sham acupuncture. | CRP was assessed within 24 h after PCI and after the 2-week intervention. MACE was assessed at 2, 4, and 8 weeks; the composite included death, malignant arrhythmia, severe heart failure, recurrent myocardial infarction, repeat PCI, and chest pain (4/16 vs 12/16). |
| Zhan et al. (33) | Both groups received the same standardized intravenous general anaesthesia; the intervention group additionally received electroacupuncture. | EA at ST36, PC6, GB34, GB24, and ST30 in one perioperative session, 30 min; no sham procedure. | CRP, IL-6, TNF-α, and ICAM-1 were assessed before surgery and on postoperative days 1 and 3. No MACE reported. |
| Ling (34) | Both groups received aspirin, clopidogrel or ticagrelor, and metoprolol; ACEI, ARB, CCB, or ARNI therapy was used as clinically indicated. | EA at PC6, HT6, and ST36, once daily, 30 min/session for 5 days. The sham group used blunt non-penetrating needles at the same acupoints, with simulated electroacupuncture but no current. | CRP, IL-6, TNF-α, IL-10, and IL-1β were assessed before PCI and after the 5-day treatment course. No MACE reported. |
| Li (35) | All groups received antiplatelet therapy, a beta-blocker, an ACEI/ARB/ARNI, and a statin. Nitroglycerin 0.5 mg or Suxiao Jiuxin pills (5-10 pills) were available as rescue medication. | TEAS at PC6 and ST36, three times/week, 20 min/session for 12 weeks. The quantitative synthesis used TEAS plus conventional treatment versus conventional treatment. | hs-CRP was assessed at baseline, weeks 4, 8, and 12, and during follow-up. No MACE reported. |
| Zeng (36) | Both groups received aspirin 100 mg/day, clopidogrel 75 mg/day, low-molecular-weight heparin, atorvastatin 20 mg nightly, metoprolol succinate extended release, isosorbide mononitrate, irbesartan, and nitroglycerin as needed; doses were adjusted where specified. | EA at PC6, HT6, SP10, LR3, SP6, and ST36, once daily, 20 min/session for 2 weeks; no sham procedure. | CRP and lipids were assessed before and after 2 weeks. No MACE reported. |

NR, not reported. Only information available in the source reports is presented; no unreported treatment or assessment details were inferred.

# 3 Supplementary Figures

**Supplementary Figure 1** hs-CRP sensitivity analysis.

**Supplementary Figure 2** CRP sensitivity analysis.

**Supplementary Figure 3** IL-6 sensitivity analysis.

**Supplementary Figure 4** TNF-α sensitivity analysis.

**Supplementary Figure 5** LDL-C sensitivity analysis.

**Supplementary Figure 6** ECG response sensitivity analysis.
